# Infection of *Ixodes ricinus* by *Borrelia burgdorferi* sensu lato in peri-urban forests of France

**DOI:** 10.1371/journal.pone.0183543

**Published:** 2017-08-28

**Authors:** Axelle Marchant, Alain Le Coupanec, Claire Joly, Emeline Perthame, Natacha Sertour, Martine Garnier, Vincent Godard, Elisabeth Ferquel, Valerie Choumet

**Affiliations:** 1 Centre National de Référence des *Borrelia*, Institut Pasteur, Paris, France; 2 Institut Pasteur – Bioinformatics and Biostatistics Hub – C3BI, USR 3756 IP CNRS –Bioinformatique et Biostatistique, Paris, France; 3 CNRS-UMR7533/LADYSS, Université de Paris 8 - Saint-Denis, France; 4 Unité Environnement et Risques Infectieux, Institut Pasteur, Paris, France; University of Maryland, College Park, UNITED STATES

## Abstract

Lyme borreliosis is the most common tick-borne disease in the northern hemisphere. In Europe, it is transmitted by *Ixodes* ticks that carry bacteria belonging to the *Borrelia burgdorferi* sensu lato complex. The objective of this work was to explore eco-epidemiological factors of Lyme borreliosis in peri-urban forests of France (Sénart, Notre-Dame and Rambouillet). We investigated whether the introduction of *Tamias sibiricus* in Sénart could alter the density of infected ticks. Moreover, the density and tick infection were investigated according to the tree species found in various patches of Sénart forest. For this purpose, ticks were sampled during 3 years. In the Sénart forest, the density of nymph and adult ticks showed no significant difference between 2008, 2009 and 2011. The nymph density varied significantly as a function of the month of collection. Regarding the nymphs, a higher rate of infection and infected density were found in 2009. Plots with chipmunks (C) presented a lower density of both nymphs and adult ticks than plots without chipmunks (NC) did. A higher rate of infection of nymphs with *Borrelia* was seen in C plots. The prevalence of the various species of *Borrelia* was also found to vary between C and NC plots with the year of the collect. The presence of chestnut trees positively influenced the density of both nymphs and adults. The infected nymph density showed a significant difference depending on the peri-urban forest studied, Sénart being higher than Rambouillet. The prevalence of *Borrelia* species also differed between the various forests studied. Concerning the putative role that *Tamias sibiricus* may play in the transmission of *Borrelia*, our results suggest that its presence is correlated with a higher rate of infection of questing ticks by *Borrelia* genospecies and if its population increases, it could play a significant role in the risk of transmission of Lyme borreliosis.

## Introduction

Lyme disease is a vector-borne zoonosis that is widespread throughout the northern hemisphere. It is caused by spirochetes belonging to the *Borrelia burgdorferi* sensu lato (sl) complex. This group includes the *Borrelia burgdorferi* sensu stricto (ss) species, as well as *B*. *afzelii*, *B*. *garinii*, *B*. *bavariensis* and *B*. *spielmanii* [1, 2]. Furthermore, there have been reports of *B*. *valaisiana* and *B*. *lusitaniae* detection in samples of human skin [3, 4]. Monitoring of the Lyme disease zoonosis is encouraged as it could be favored by global warming. The disease distribution is directly related to that of the vector, which requires a cool, moist habitat, mostly forest. In Europe, the incidence rates vary from less than 5 per 100,000 in Ireland to 300–350 per 100,000 in Austria, where the highest incidence is currently reported. In France, the incidence varies greatly depending on the region, and has been estimated as a global average of 40 per 100,000 inhabitants [5].

The lack of a notification system, and the difficulty to collect comprehensive clinical data, explain the limited data available in France concerning the exact incidence of the disease, its distribution across regions, and the predominant clinical forms. Another approach to analyze the epidemiology of the disease is to study the vector, *Ixodes ricinus*, the most common tick found in our forests and grasslands. Data including distribution, density and the rate of infection by different pathogens are an indication of the risk to the population in a given region. This analysis of the acarological risk therefore constitutes a complementary approach to epidemiological studies in humans [6, 7].

Since the mid-1990s, there is evidence that this tick is present throughout the French territory, with the exception of the Mediterranean rim and areas over 1200 m in altitude. However, wide variations exist from one region to another and even from one site to another within a given region [6, 7].

The vector of Lyme borreliosis in Europe is *Ixodes ricinus*, whose distribution extends from North Africa to Scandinavia in longitude and Ireland to European Russia in latitude [8]. During its life, the tick goes through two successive stages (larval and nymphal) before reaching the adult form.

The perpetuation of the cycle and the maintenance of bacteria in the natural environment are due to the existence of host species known as reservoirs, which can tolerate and maintain pathogens, but also effectively transmit the pathogen to ticks. The most important species as reservoirs in the cycle of *Borrelia burgdorferi* sl are small mammals, especially rodents, and birds [9–12]. These are specific hosts for some *Borrelia* species, with a particular association between *B*. *garinii* and birds or between *B*. *afzelii* and small rodents [13–17]. These preferences seem to be explained by complex interactions between the bacteria and their hosts, which particularly involve the complement system [10, 18, 19].

Our study was focused on peri-urban forests of Île-de-France. These forests are frequented by many visitors and the risk of exposure to tick bites is high. One of them, the Sénart forest, is located 30 km south of Paris (in the Île-de-France region) and has a large number of visitors (3 million per year in the late 1990s) [20]. This forest has the characteristics of being partly invaded by chipmunks (*Tamias sibiricus*). The chipmunk has been introduced from Eurasia, particularly Siberia, China and Korea. The first individuals were released by their owners at the western end of the Sénart forest, in the 1970s [21]. The northeastern part of the forest was colonized recently. Our current study aims to evaluate the evolution of the infection of *Ixodes ricinus* by *Borrelia burgdorferi* sl. by comparing the results obtained during 3 years and to determine the consequences of the proliferation of this non-native rodent species, *Tamias sibiricus*, on the risk of transmission of Lyme borreliosis. For this purpose, we analyzed the rate of infection and the density of infected ticks during 2008, 2009 and 2011 in several locations of the Sénart forest. These results were compared to those obtained for ticks collected in 2009 in two other peri-urban forests of Île-de-France (Rambouillet and Notre-Dame) that have not yet been colonized by these rodents. Although we found a high rate of infection in plots with chipmunks in the Sénart forest, our results show only a significant difference in 2011 in terms of the density of infected nymphs between locations colonized or not by *Tamias sibiricus*.

## Material and methods

### Ethics statement

A permission to circulate in the various forests was required to the Office National des Forêts. No specific permission was necessary for tick collection.

Field studies did not involve endangered or protected species. *Tamias sibiricus* is an invasive exotic species of concern at European level and does not require any permission for collection and tick sampling.

### Sites of tick collection

**Fig 1:**
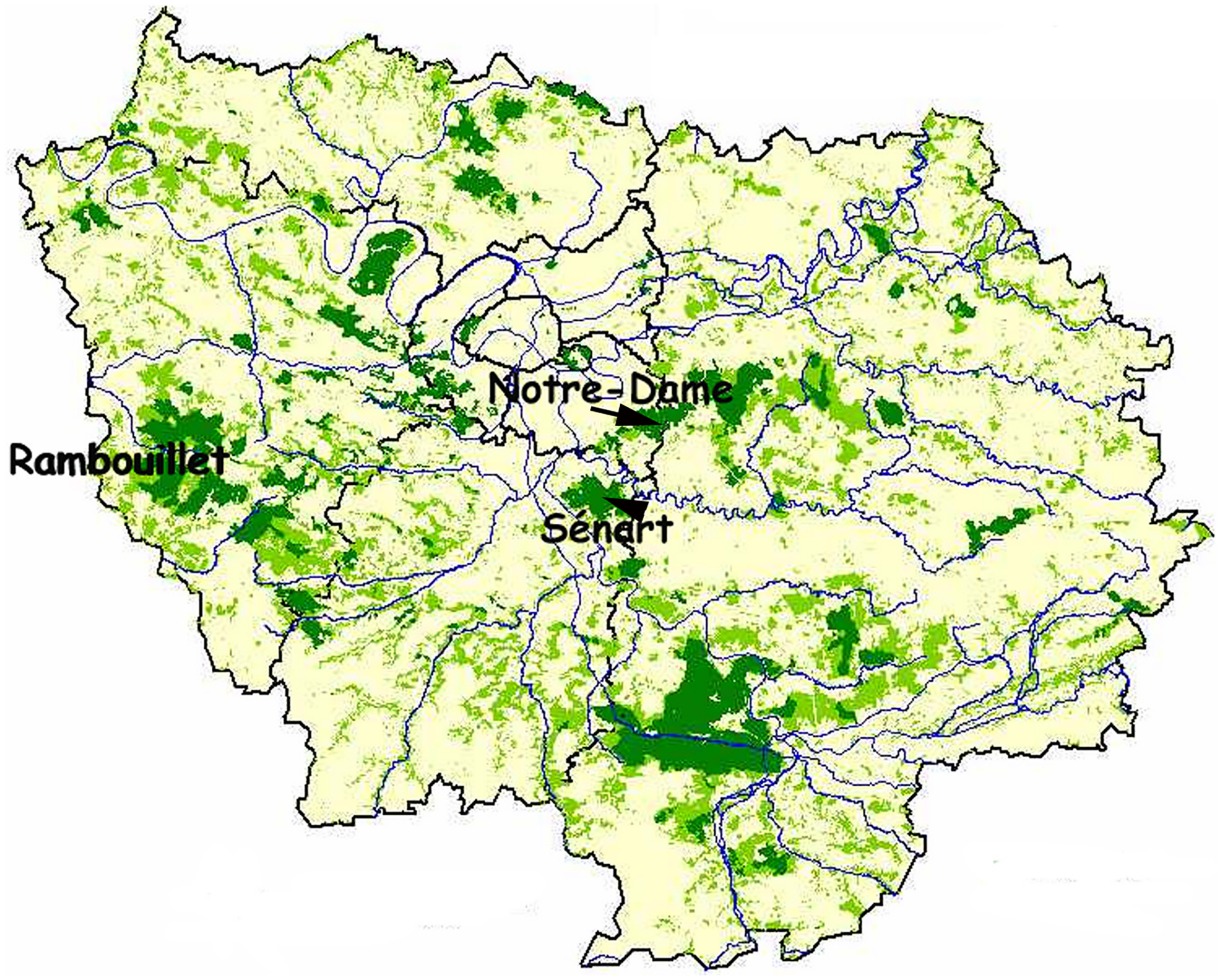
Location of the three forests studied.

To assess the role of *Tamias sibiricus* in the risk of Lyme disease transmission, we performed a longitudinal study from March to October during three years (2008–2009 and 2011) in the Sénart forest and from April to October 2009 in Rambouillet and Notre-Dame forests (Fig 1). To select precisely the stations to study, we relied on 1/25000 maps published by Institut Géographique National (IGN) that were cut into 100m by 100m plots using Photoshop software. Eight locations differing in the presence/absence of chipmunks were chosen at random in the Sénart forest.

#### Sénart forest

**Fig 2:**
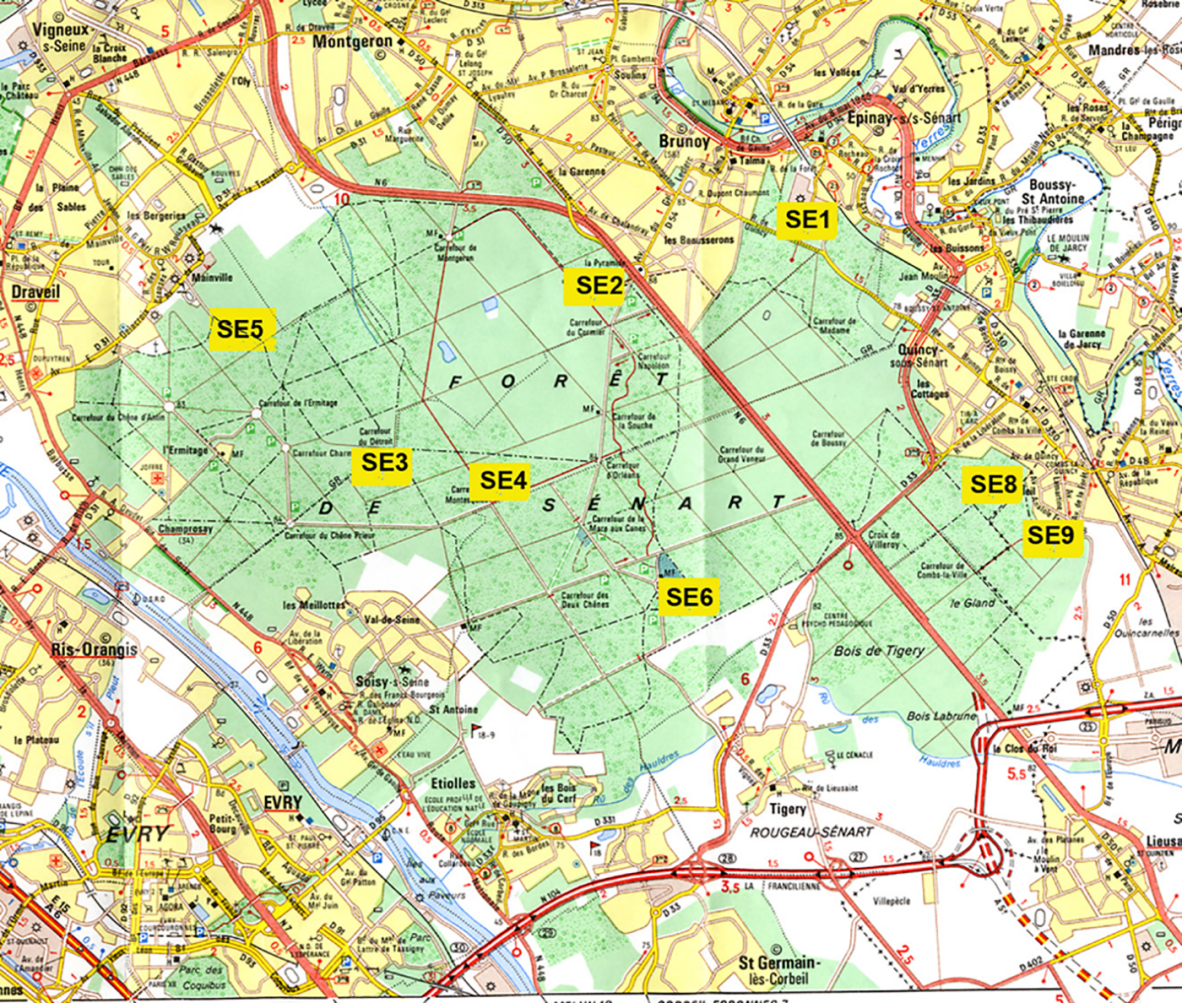
Location of the plots where ticks were collected in the forest of Sénart.

The Sénart forest (3156 ha) is located in the suburban area, 22 km south of Paris in the Essonne department. The main tree species are oak, chestnut, hornbeam, birch, Scots pine and other conifers. Depending on the plot, the distribution of the main tree species is different (S1 Table). The fauna consists in wild boar, roe deer, foxes, hares, rabbits and squirrels. Among the bird species found in the forest are pigeons, woodcocks, crows, ducks, magpies, blackbirds and jays. 8 plots (SE1, SE2, SE3, SE4, SE5, SE6, SE8 and SE9) were drawn randomly using Epi Info software (Fig 2) (S1 Table). In the field, the stations are found by GPS. Chipmunks were identified in plots SE2 to SE6, few were seen in plot SE1 whereas plots SE8 and SE9 are not invaded yet by these rodents.

#### Rambouillet and Notre-Dame forests

Rambouillet forest is one of the main forests of Île-de-France. It is a wooded area of 14 550 ha. The tree population consists mainly of oak, up to 68%, and conifers (Scots pine and Corsican pine) to 25%. The large mammal fauna is composed of deer, roe deer and wild boar and among the small mammals, we find the hare, rabbit, European badger, fox, weasel, marten, skunk, dormouse, wood mouse, bank voles, shrews, moles, squirrels and hedgehogs. Many bird species benefit from different habitats provided by the forest, including a number of birds protected at European level as the black woodpecker, the red-backed shrike species, woodpecker, little bittern, the nightjar of Europe and the honey buzzard. 8 plots (R2, R5, R6, R7, R8, R10, R17 and R20) were drawn randomly using Epi Info software (S1 Table).

The Notre-Dame forest (2050 ha) is composed of chestnut (60%); oaks (11%); birch (11%); acacia (10%); other deciduous trees (6%); conifers (2%). It has a rich and diverse fauna and has two types of large animals involved in the life of the ecosystem, wild boars, and roe deer. Small mammals (bank voles), birds (*Turdus* sp.) as well as amphibia were also described. Two plots (ND10 and ND11) were drawn randomly using Epi Info software (S1 Table).

### *Ixodes ricinus*’ sampling

Tick sampling was achieved through the flag method that collects the questing ticks [7]. The flag method is considered pertinent to develop a statistically rigorous, objective methodology [22]. This method uses a lure that mechanically simulates a host’s passage. The collector drags a square of fabric measuring 1m^2^ over a distance of 10 meters at a rate of 50cm per second, in order to collect ticks in an area of 10 m^2^. A total of 16 surveys are conducted per hectare of land to randomly explore 160m^2^ per station. Only adults and nymphs were collected, larvae were merely counted because transovarial transmission is very low [23]. Temperature, hygrometry and time of collect were recorded every month on each plot.

#### *Ixodes ricinus’* density

Ticks’ density (d), expressed in number of ticks per 100 m, is estimated from the total collected ticks’ number according to [7].

#### Infection rates

The ticks’ infection rate is calculated using the following formula:

p=f / k

f: number of ticks infected with *B*. *burgdorferi* sl in each plot

k: number of ticks analyzed in each plot

#### Data comparison

**Bivariate analysis:** Using the software Statistica, we could test by the Kolmogorov test, the normality of data density and by the Brown-Forsythe test, the homoskedasticity. Since the values did not follow a normal distribution, or because homoskedasticity was not verified, then we applied a Kruskal-Wallis followed by Mann Whitney test, taking into account the Bonferroni correction.

To compare infection rates, we use the χ ^2^ test (Chi2) Mantel-Haenszel or Fisher's exact test, calculated using Epi-Info. The correlation between the data is made using the Spearman test.

**Multivariate analysis:** We performed a principal component analysis (PCA), which is a popular dimension reduction method to visualize data. PCA aims at compressing the size of the data in order to extract the most important information. This method offers a simplification of the data while analyzing the structure of both observations and variables [24]. A log10 transformation was applied on the 4 density variables (infected/non infected, nymph/adult) as it increases the inertia of the first 2 principal components leading to a better visualization of the dataset. Note that applying a monotonically increasing transformation such as log10 does not affect the interpretation of correlations.

In this article, the widely used scree rule [25] that was applied to determine the number of components to retain. PCA was performed using the open source statistical software R and the R package FactoMineR that provides a tool to perform and visualize results of PCA. The few missing data were imputed using the R package missMDA that performs multiple imputation using multivariate data analysis. The principal components are computed from active variables, meaning the 4 density variables (infected/non infected, nymph/adult), the 2 infection rates (nymph, adult), the percentages of chestnuts, oaks and pines, temperature, hygrometry and hour. Therefore, the year and the qualitative variables, meaning the month, the chipmunk status and the plot number are projected as supplementary variables on the factorial map.

### Chipmunks analysis

Eighty-one chipmunks were captured in September 2008. We extracted 81 ear samples that were analyzed by PCR and RFLP (Restriction Fragment Length Polymorphism) and 60 were placed in culture. All thermo lysates were analyzed by PCR and RFLP if necessary to identify the *B*. *burgdorferi* sl. species. If the RFLP did not allow clear conclusions to be drawn, then the DNA was sequenced.

The chipmunk density was evaluated during tick collection in the various plots of Sénart. In SE2, SE3, SE4, SE5 and SE6 plots, an average of 4 chipmunks were observed from May to September during 1h30. In SE1 plot, only 1 chipmunk was observed during 1h30 of collection, whereas no chipmunk was observed in SE8 and SE9 during the same time of collect (Fig 2).

### Pathogen detection and identification

Nymph numbers collected by plots can be very large (over 1000 nymphs collected in June), and laboratory techniques are heavy. We therefore analyzed a sample of 30 nymphs and 26 adults randomly per plot which corresponds to the minimum numbers required for statistical analyzes.

#### DNA extraction

After sampling, ticks were kept alive until analysis [7]. We used a buffer prepared with 5 ml of Tris-HCl (1M, pH 8), 0.5 ml of Tween 20, 200 ml of EDTA (1M, pH 8) and 94.3 ml of distilled water. Knowing that *Borrelia* are, for most of them, located in the gut of the tick, we used fine needles, each tick was lacerated individually in lysis buffer containing proteinase K (for nymphs 2μl of proteinase K was added to 50μl of buffer and twice as much for adults) under a binocular microscope. Then, tubes containing lysis buffer and dilacerated ticks were placed in a water bath at 56°C for 4 hours minimum to lyse the cells and release the DNA, then at 100°C for 10 min to denature the proteinase K. The samples were then cooled on ice and centrifuged at 14000 rpm. The extracts obtained were stored at 4°C before being analyzed by PCR. For long term storage, the samples were stored at -20°C.

#### Pathogen DNA detection by polymerase chain reaction (PCR)

We performed a gene amplification reaction to detect the presence of the *Borrelia* DNA. To identify *B*. *burgdorferi* sl, several genomic DNA fragments or plasmid can be amplified [26–28]. As part of our study, the selected region corresponds to the intergenic spacer between the gene encoding subunits 5S (*rrf*) and 23S (*rrl*) of ribosomal RNA. This is a single space in bacteria, encoding the ribosomal RNA, with a tandem repeat of 5S and 23S genes specific for the *B*. *burgdorferi* sl complex [29]. Primers used to amplify this segment are the following: forward 5’GAAAAGAGGAAACACCTGTT3’ and reverse 5’TCGGTAATCTTGGGATCAAT3’ at 56°C. In case of doubtful or weak signals, a nested PCR was performed as described in [6] using the following primers: forward 5’ CTGCGAGTTCGCGGGAGAG’ and reverse 5’ TCGGTAATCTTGGGATCAAT3’ at 64°C.

#### *Borrelia burgdorferi* sensu lato species identification by restriction fragment length polymorphism (RFLP)

Complex *B*. *burgdorferi* sl species identification is based on the analysis of RFLP. Positive PCR products following *rrf-rrl* intergenic spacer amplification were digested with the restriction enzyme MseI and analyzed by electrophoresis [30]. According to D. Richter et al. [31], the rrf-rrl intergenic spacer is a good locus for *Borrelia* taxonomy because it varies widely.

The DNA corresponding to the *Borrelia* restriction profiles that are difficult to identify were sent to be sequenced in order to perform multiple alignments of the sequences obtained with sequences of reference strains using Clustalw. Distances were calculated with Jukes and Cantor correction. From these alignments, a tree was constructed with UPGMA in MEGA 3.1 software.

### Tree species in Sénart

We used records of vegetation provided by The Office National des Forêts (ONF) in the studied plots. Different plant species were expressed as percentages for the eight plots analyzed in our study. Moreover, We then tested with a Spearman test if there was a correlation between the frequency of the different species in the 8 plots and various parameter related to *I*. *ricinus* (density, rate of infection, density of infected ticks) from different plots. We considered that the Spearman correlation was moderate with coefficient R values ranging from 0.4 to 0.59 and significant when R was above 0.6 (or less than -0.6).

## Results

### Collection of ticks: Analysis of total density, infection rate and density of infected ticks

#### Sénart forest

In the Sénart forest, 28529 nymphs and 1302 adult ticks were collected in 2008, 2009 and 2011 from March to October. 4973 nymphs and 1226 adults were analyzed. The density of nymphs and adults showed no significant difference between the three years of tick collection (Table 1).

**Table 1:** Density and infection status of ticks *I*. *ricinus* in the forest of Sénart in 2008, 2009 and 2011 on eight plots.

	2008	2009	2011	Statistics	Total
**Nymphs (N)**	7162	8701	12666	NS	28529
**Nymph density/100 m**^**2**^	69.9	85.2	126.9		94
**Adults (N)**	407	495	400	NS	1302
**Adult density/100 m**^**2**^	4	4.9	4		94
**Nymph Infection rate**	165/1650	215/1682	139/1641	**P<0.0002 2009>2011 = 2008**	519/4973
**Nymph Infection rate** %	10	12.8	8.5		10.4
**Adult Infection rates**	37/390	52/473	34/363	NS	123/1226
**Adult Infection rates** %	9.5	11	9.4		10
**Density of infected Nymphs/100 m**^**2**^	7	11	10.7	**P<0.024 2009>2008**	9.8
**Density of infected Adults/100 m**^**2**^	0.4	0.5	0.4	NS	0.4

NS: non-significant; N: number of nymphs and adult collected

The density of nymph showed a significant difference according to the month of collect in 2008, 2009 and 2011, whereas no difference was observed for adults (S2 Table). Regarding the infection rate of nymphs, a significant difference (p<0.0002) was found between the years, 2009 being higher than 2008 and 2011. The density of infected nymphs was significantly higher in 2009 than in 2008 (p<0.024). Density of infected adult ticks showed no difference according to the year of the collect.

**Fig 3:**
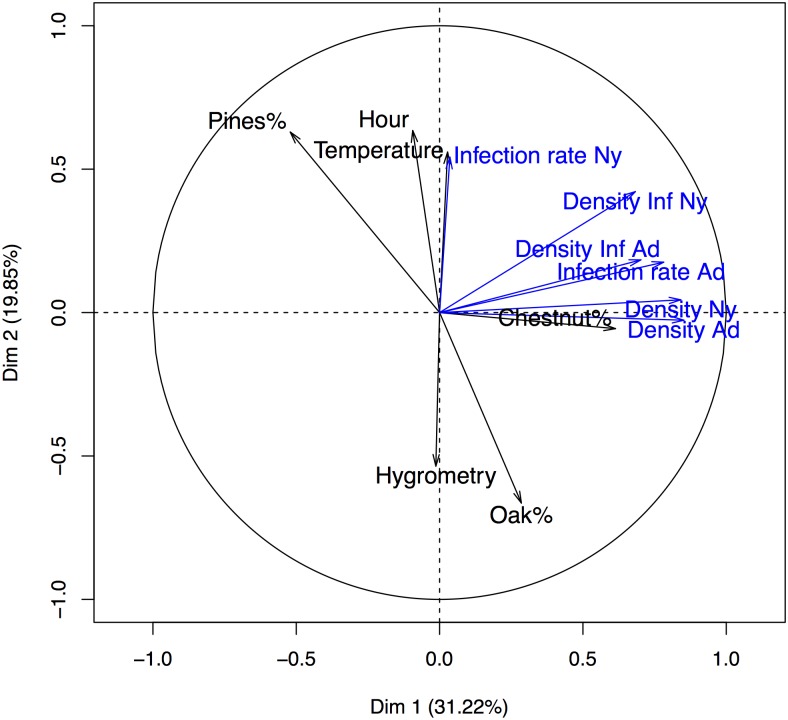
Graph of variables computed by PCA. The tick variables (density, infection rate, density of infected ticks) are presented in blue while in black tree species and climatic data are represented.

We performed a PCA analysis in order to extract the most important information. The elbow rule suggests retaining 2 dimensions, which explain 31.22% of inertia for first axis and 19.85% for second axis. This means that 51.07% of the information contained in the data can be summarized on a 2 dimensions map. According to the correlation circle displayed in Fig 3, two groups of correlated variables are discerned: the first dimension discriminates observations with larger values for densities, infection rate of adults and percentage of chestnuts on the right of the factorial map.

**Fig 4:**
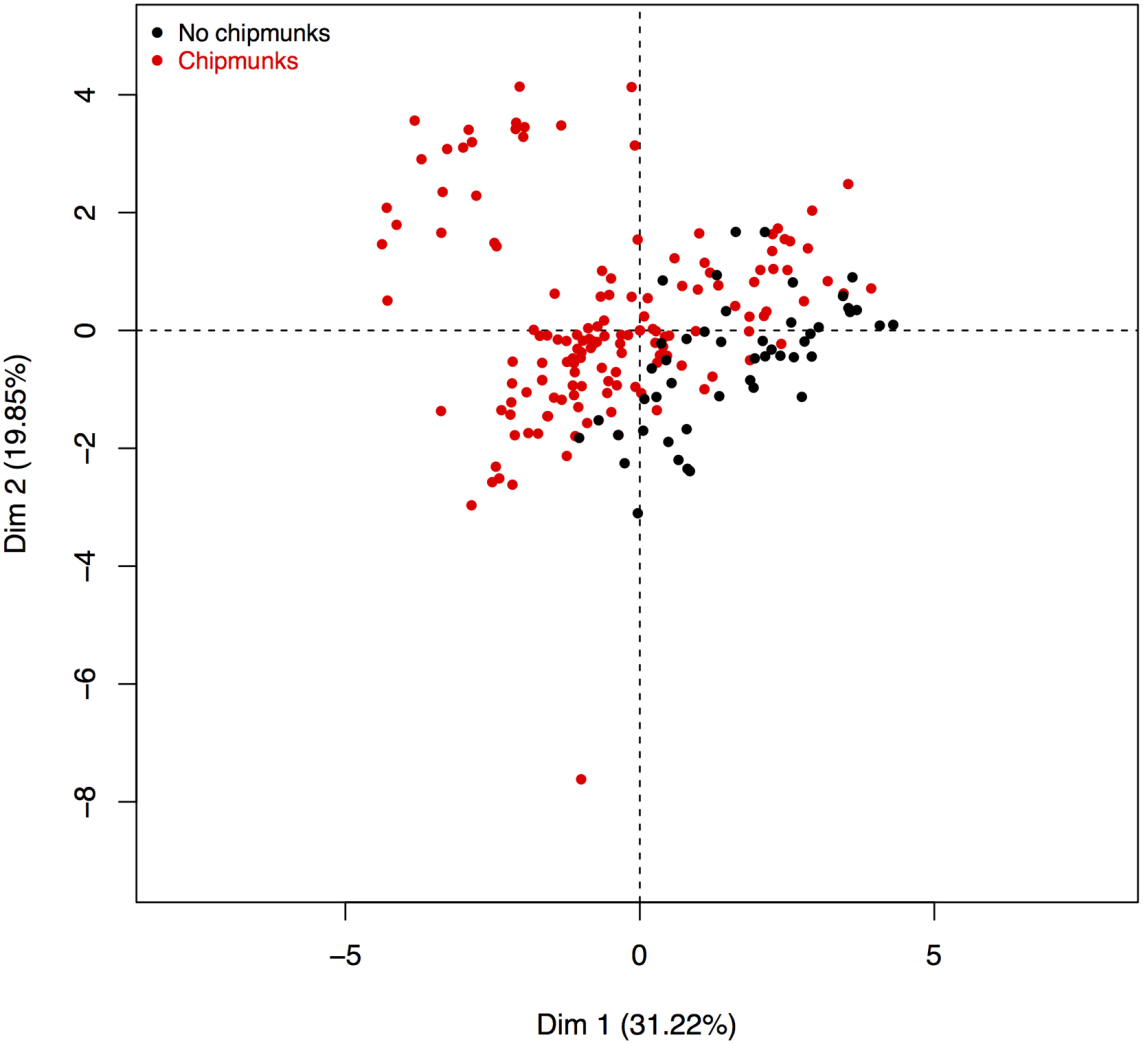
Factorial map of PCA. The dots are colored according to the chipmunk status (in black: plot with no chipmunk, in red, plots with chipmunks).

The correlation matrix displayed in S3 Table gives more insight about correlations among this collection of variables: variables characterizing the first dimension of PCA are positively correlated. This means that observations with positive coordinates on the first dimension exhibit larger values for this group of variables (right panel of the Fig 3). In the same manner, hour, temperature, hygrometry, percentages of oaks and pines and infection rate of nymphs contribute most to dimension 2. The S3 Table highlights that hour and temperature are positively correlated while temperature and hygrometry are negatively correlated which is rather consistent. Fig 4 and S1 and S2 Figs display the scatter plot of observations, in the factorial map. The dots are colored according to the chipmunk status (Fig 4), year of the experiment (S1 Fig) and month (S2 Fig).

On Fig 4, one can notice that the first dimension efficiently discriminates the observations according to the chipmunk status of the corresponding plots. This figure also suggests that the absence of chipmunks and larger percentages of chestnuts are associated to larger values of densities (infected or not) of nymphs and adults and infection rate of adult ticks. The scatter plots of S1 and S2 Figs show that no cluster of years or months is observed that suggests that plots are homogenous.

#### Rambouillet and Notre-Dame forests in 2009

In the Rambouillet and Notre-Dame forests in 2009, respectively, 4587 and 941 nymphs and 261 and 49 adults were collected between April and October. 1234 and 326 nymphs and 239 and 46 adults were analyzed respectively for each forest. The density of nymphs and adult ticks, their rate of infection and the density of infected ticks according to the month of collection in these 2 forests were compared to the same parameters in the Sénart forest over the same time period (S4 Table).

The nymph and adult densities were similar in Sénart, Notre-Dame and Rambouillet forests (S4 Table) while the infection rate of nymphs was significantly higher in Sénart than in Rambouillet (p<0.002) but was similar to Notre-Dame. The density of infected nymph was higher in Sénart than in Rambouillet and Notre-Dame whereas the infected adult densities did not differ significantly, regardless of the forest.

### Influence of *Tamias sibiricus* in the Sénart forest

We first compared six plots on which chipmunks are present (C plots: SE1, SE2, SE3, SE4, SE5 and SE6) and two plots without chipmunks (NC plots: SE8 and SE9). These plots were further subdivided in three series according to chipmunk abundance: α plots on which the chipmunks are present in large numbers (SE2 to SE6), with β plot (SE1) with few chipmunks and γ plots without chipmunks (SE8 and SE9) (Fig 2). We also compared these results to those obtained in Rambouillet and Notre-Dame where this non-native species has not been introduced.

#### Comparison of tick parameters in C and NC plots

We observed a difference in the density of nymphs and adults for all years, C plots having a significantly lower density than NC plots (Table 2). Infection rates of nymphs were higher in C plots whatever the year of collection while that of adults was only significantly higher in 2009. Density of infected nymphs differs significantly between NC and C plots only in 2011, and in 2008 and 2011 for adults, NC plots presenting a higher density than C plots (Table 2).

**Table 2:** Role of chipmunks on the density and infection of *I*. *ricinus* ticks in the forest of Sénart in 2008, 2009 and 2011.

	2008 C plots	2008 NC plots	Statistics 2008 C/NC	2009 C plots	2009 NC plots	Statistics 2009 C/NC	2011 C plots	2011 NC plots	Statistics 2011 C/NC	Statistics 2008-2009-2011 C plots	Statistics 2008-2009-2011 NC plots
**Density of Nymphs /100 m**^**2**^	55.5	113.4	p<0.0009 **NC> C**	62.8	152.3	p<0.011 **NC> C**	67.7	300.8	p<0.0000 **NC> C**	NS	**p<0.002**
											2008 = 2009
											2008<2011
											2009<2011
											2011>2009 = 2008
**Density of Adults /100 m**^**2**^	2.2	9.3	p<0.0000 **NC> C**	3.2	9.8	p<0.00001 **NC> C**	1.6	10.9	p<0.0000 **NC> C**	NS	NS
**Nymph infection rates**	12.1%	4.8%	p<0.0000 **C>NC**	14.5%	8.5%	p<0.0001 **C>NC**	9.7%	5.6%	p<0.003 **C>NC**	**p<0.002**	NS
										2009>2011	
										2009 = 2008	
										2008 = 2011	
**Adult infection rates**	11.3%	8.2%	NS	14.8%	7.4%	p<0.01 **C>NC**	10.1%	9%	NS	**NS**	NS
**Density of infected Nymphs/100 m**^**2**^	6.7	5.4	NS	9.1	13	NS	6.5	16.8	p<0.026 **NC> C**	**NS**	**p<0.0057**
											2008<2009
											2008<2011
											2009 = 2011
											2011 = 2009>2008
**Density of infected Adults/100 m**^**2**^	0.2	0.8	p<0.002 **NC> C**	0.5	0.7	NS	0.2	1	p<0.0021 **NC> C**	**NS**	**NS**

NS: non-significant

The two series of plots were compared between years. For C plots, we only observe a difference in the rate of infection of nymphs (p<0.002), 2009 being higher than 2011. For NC plots, the density of nymphs was significantly different according to the year (p<0.002), 2011 being higher than 2009 and 2008). Density of infected nymphs was also significantly different (p<0.006); 2008 being lower than 2011 and 2009.

#### Comparison of tick parameters between α, β and γ plots

**Sénart forest:** S3 Fig shows that PCA analysis supports the subdivision of plots with no (α), few (β) and with chipmunks (γ). We then compared globally the density of the nymphs and adult ticks collected on α, β and γ plots in 2008, 2009 and 2011. A significantly higher density was observed in β and γ plots compared to α plots for both nymphs and adults (p < 0.0001) (S5 Table). The infection rate of nymphs was shown to vary significantly whatever the year, that of γ plots being the lowest. For adults, a significant difference was observed only in 2009, the rate of infection in γ plots being lower than in β plot. In 2008 and 2011, the density of infected nymphs in α plots was inferior to β plot. Regarding the adults, a significantly higher infected density was found in β and γ plots in 2008 and 2009 compared to α plots.

We compared also the density of nymphs and adults in each group of plots according to the year of collect. We found only a difference on γ plots, for which the density of nymphs was higher in 2011 (p<0.002) (S5 Table).

The rate of infection of nymph shows only a difference on α plots, which was higher in 2009 than 2011 (p<0.002) (S5 Table). For adults, no difference was observed in the various series of plots whatever the year.

The density of infected nymphs showed a difference on γ plots, which was lower in 2008 (p<0.006).

**Fig 5:**
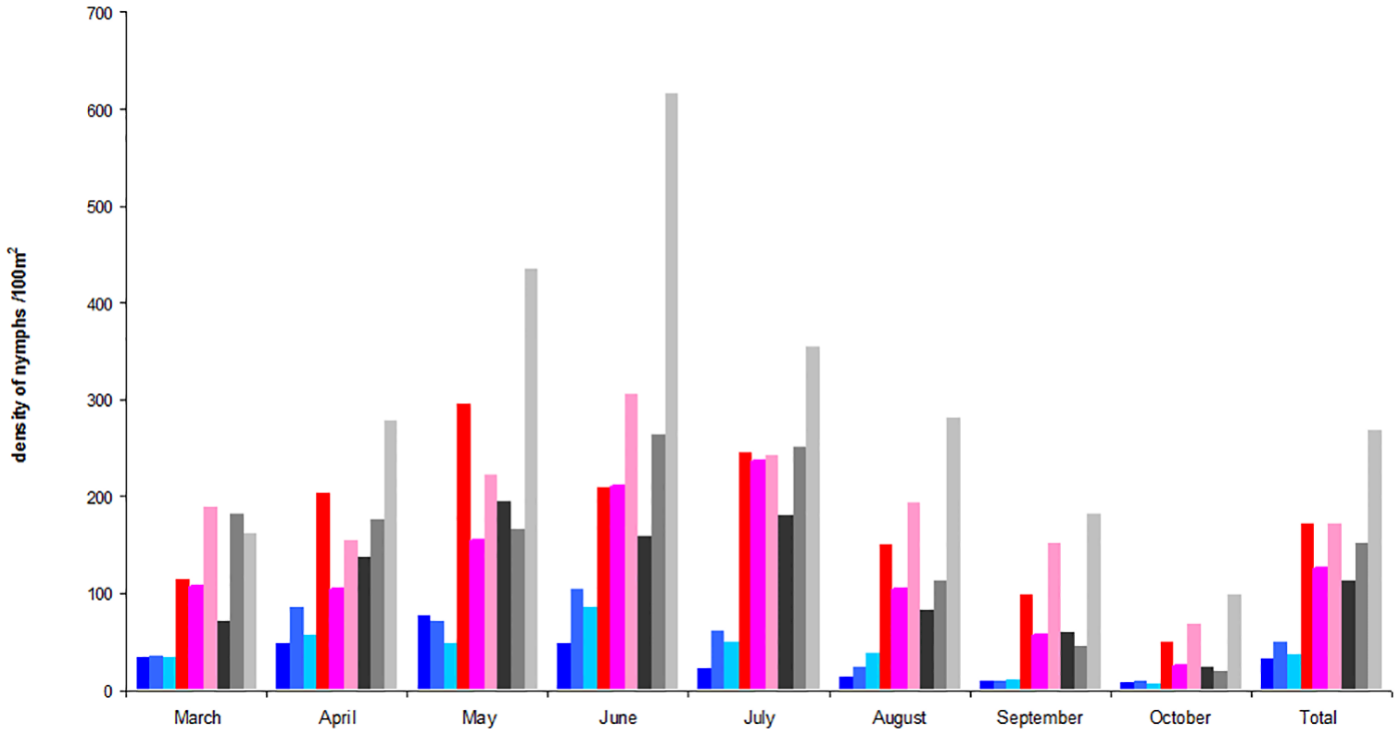
Density of *I*. *ricinus* nymphs in the five plots with chipmunks (α), one with little chipmunk (β) and two plots without chipmunk (γ) from the forest of Sénart in 2008, 2009 and 2011. α plots in 2008 (dark blue square), α plots in 2009 (cobalt blue), α plots in 2011 (aquamarine square); β plots in 2008 (red square), β plots in 2009 (fuchsia square), β plots in 2011 (pink square); γ plots in 2008 (black square), γ plots in 2009 (grey square), γ plots in 2011 (light grey square).

**Fig 6:**
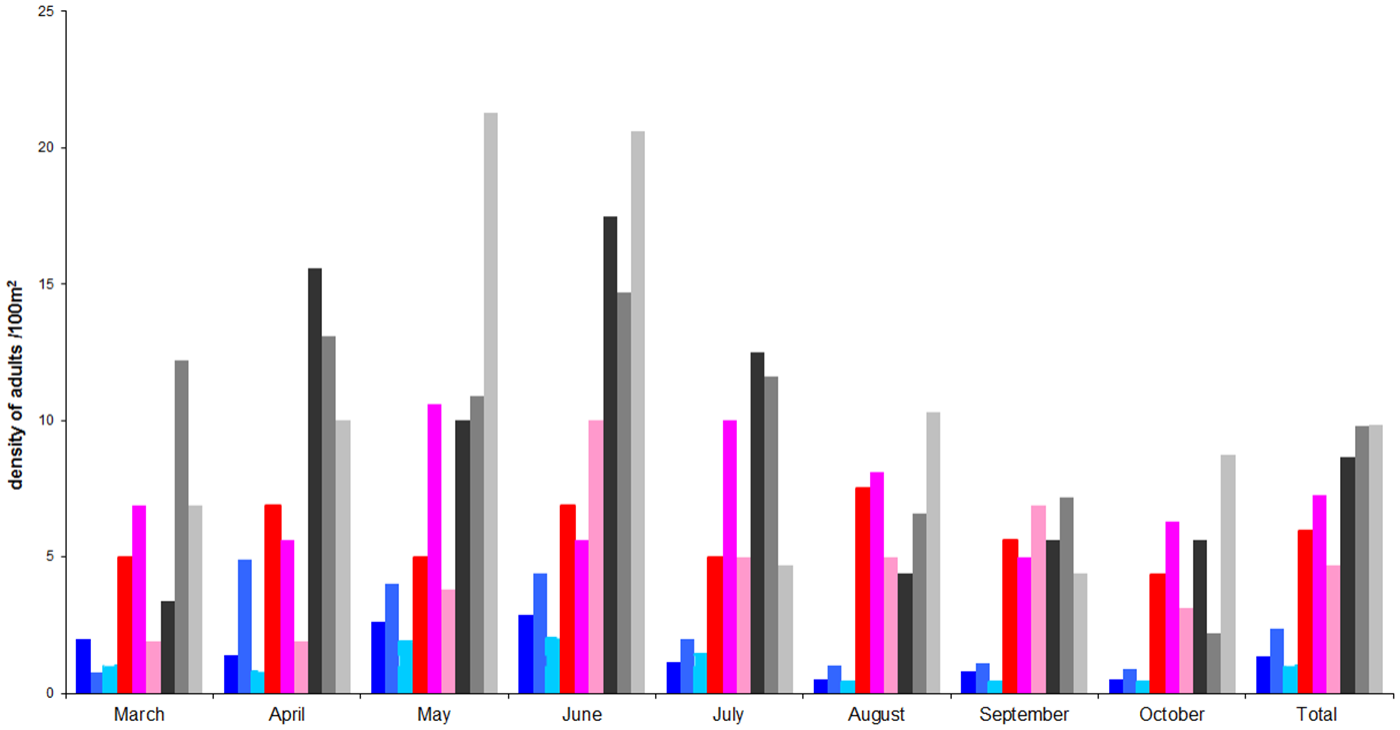
Density of *I*. *ricinus* adults in the five plots with chipmunks (α), one with little chipmunk (β) and two plots without chipmunk (γ) from the forest of Sénart in 2008, 2009 and 2011. α plots in 2008 (dark blue square), α plots in 2009 (cobalt blue square), α plots in 2011 (aquamarine square); β plots in 2008 (red square), β plots in 2009 (fuchsia square), β plots in 2011 (pink square); γ plots in 2008 (black square), γ plots in 2009 (grey square), γ plots in 2011 (light grey square).

The density of nymphs and adults was grouped during the three years of collect and compared according to the month of collect for each group of plots (Figs 5 and 6). We only found a significant difference in α plots (p<0.0001), for which the density of nymphs in August, October and November was lower compared to that of the other months. The same comparison was performed for adult ticks and did not provide any significant difference whatever the month of collect and the group of plots studied.

### Comparison of Sénart forest with Rambouillet and Notre-Dame forests in 2009

When these data were compared to those obtained in the other forests studied during the same months of 2009, the adult and nymph density was significantly higher (p <0.0001 and p<0.02 for adult and nymphs respectively) in NC plots of Sénart (SNC) compared to Rambouillet and Notre-Dame forests (Table 3). The adult infection rate is higher in Rambouillet than in SNC plots (p<0.004). The density of infected nymphs was higher in SNC plots than in Rambouillet and Notre-Dame (p<0.019).

**Table 3:** Comparison of characteristics of *I*. *ricinus* ticks in the forests of Sénart (C and NC plots), Rambouillet and Notre-Dame in 2009.

	Sénart C plots	Sénart NC plots	Rambouillet	Notre-Dame	Sénart C plots Rambouillet Notre-Dame	Sénart NC plots Rambouillet Notre-Dame
**Density of Nymphs/100 m**^**2**^	56.9	154	60.6	49.1	NS	**p<0.02**
						**SNC>R = ND**
**Density of Adults/100 m**^**2**^	2.9	10	3.5	2.6	NS	**p<0.0001**
						**SNC>R = ND**
**Infection rate of Nymphs**	15%	9%	8.8%	9.8%	**<0.000007**	NS
					**SC>ND = R**	
**Infection rate of Adults**	14.9%	7%	17.6%	8.7%	NS	**p<0.004**
						**R>SNC**
**Density of infected Nymphs/100 m**^**2**^	9	14.1	5.3	4.8	NS	**p<0.019**
						**SNC>R = ND**
**Density of infected Adults/100 m**^**2**^	0.3	0.7	0.6	0.2	NS	NS

SNC SNCNS: non-significant; SNC: NC plots of Sénart; R: Rambouillet; ND: Notre-Dame

We did not find any difference in the density of adults and nymphs between C plots of Sénart and plots of the two other forests. The nymph infection rate of the C plots of Sénart forest was higher than for the nymphs collected in Rambouillet and Notre-Dame forests (p <0.0001), whereas a similar rate of infection was observed for adults in the forests of Rambouillet, Notre-Dame and the C plots of the Sénart forest (Table 3).

### Infection of ticks in the different forests by *Borrelia* genospecies

A total of 36047 ticks were collected from 2008, 2009 and 2011 in the forests of Sénart, Notre-Dame and Rambouillet and 8253 ticks were analyzed. We first analysed the polymorphism of the *rrf-rrl* spacer after digestion with *Mse*I. The patterns obtained allowed various strains belonging to various groups in several species to be identified.

#### Sénart forest

In 2008, 2009 and 2011, *B*. *afzelii*, *B*. *burgdorferi* ss and *B*. *garinii* were the most prevalent species identified in nymphs, with variations according to the year of collect. Regarding the comparison between C and NC plots, again the same three species were prevalent with variations only for *B*. *afzelii* in 2011 and for *B*. *burgdorferi* ss in 2008 (C<NC) (S6 Table). When α, β and γ plots were compared, again these species were the most prevalent with variations observed according to the year for *B*. *burgdorferi* ss and *B*. *garinii*. Interestingly, *B*. *lusitaniae* were only identified in α plots, whatever the year of collect (Table 4).

**Table 4:** Prevalence of *Borrelia* species in nymphs collected in 2008, 2009 and 2011 in α, β and γ plots of the Sénart forest.

Nymphs	2008 total plots %	2009 total plot %	2011 total plots %	Statistics years	2008 2009 2011 α plots %	2008 2009 2011 β plots %	2008 2009 2011 γ plots %	2008 2009 2011 total plots %	Statistics α / β / γ
***B*. *afzelii***	32	**47**	**21**	p<0.000001	36	34	33	35	NS
				2009>2008					
***B*. *burgdorferi ss***	27	30	**30**	NS	**29**	15	**37**	29	p<0.008
									γ = α > β
***B*. *garinii***	**21**	10	**21**	p<0.005	14	**34**	13	17	p<0.000015
				2008 = 2011>2009					β > α = γ
***B*. *lusitaniae***	4.2	0.5	**9.4**	p<0.0002	5.8	0	0	4	p<0.0
				2011>2009					
				2011 = 2008					α > γ
				2008 = 2009					
***B*. *spielmanii***	1.8	1.4	2.2	NS	1.4	0	4.4	1.7	NS
***B*. *valaisiana***	12	5.6	12	NS	9.4	12	6.6	9.2	NS
**Co-infection**	2.4	6	4.3	NS	4.2	4	5.5	4.4	NS
**Percentage of infected ticks**	10	**12.8**	8.5	p<0.0002	**12.84**	9.31	6.31	10.44	α > β > γ
				2009>2011 = 2008					
**Statistics**	***Ba = B*.*b*ss = *B*.*g***	***Ba>Bb*ss>*Bg***	***Bb*ss = *Bg* = *Ba***		***B*.*a = B*.*b*ss>*B*.*g***	***B*.*a = B*.*g>B*.*b*ss**	***B*.*b*ss = *Ba>B*.*g***	***Ba = Bb*ss>*B*.*g***	

NS: non-significant; *Ba*: *B*. *afzelii*; *Bg*: *B*. *garinii*; *Bb*ss; *B*. *burgdorferi* ss;

For adults, in 2008, 2009 and 2011, four species were found more prevalent: *B*. *burgdorferi* ss, *B*. *garinii*, *B*. *afzelii* and *B*.*valaisiana*. The same trend is observed on C and NC plots as well as on α, β and γ plots of Sénart (S7 Table).

#### Comparison with other forests of Île-de-France

**Fig 7:**
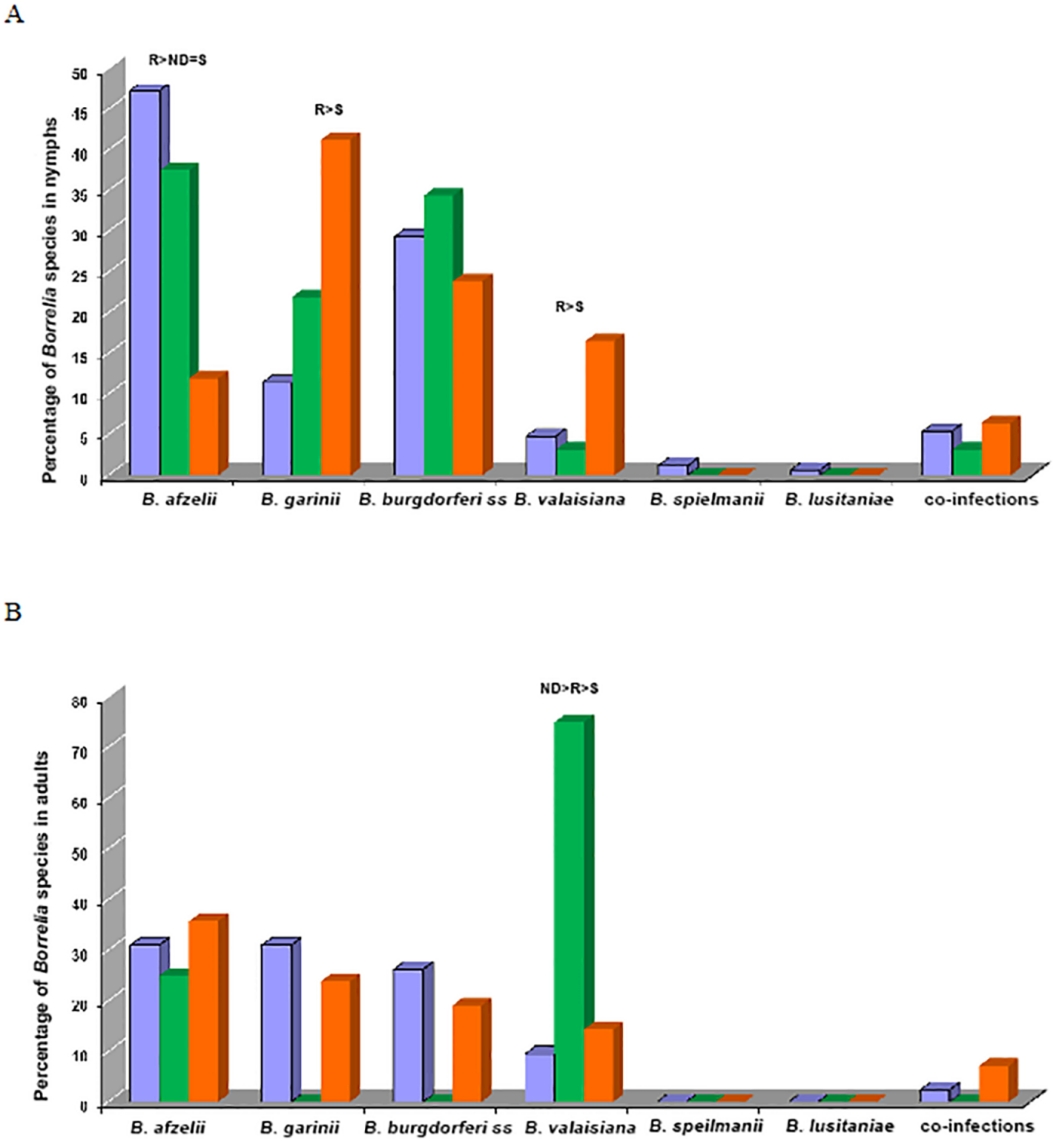
Comparison of the prevalence of the various *Borrelia* species in the peri-urban forests of Rambouillet, Notre-Dame and Sénart. ND: Notre-Dame (green square); R: Rambouillet (red square); S: Sénart (blue square). Regarding the adult ticks in 2009, no significant difference was shown in the various forests, except for *B*. *valaisiana* (p <0.002), which is prevalent in the forest of Notre-Dame (75%) (p<0.009).

We compared the results obtained at Sénart (C and NC plots were grouped since no significant difference was found between them for each species) with those obtained at Rambouillet and Notre-Dame forests in 2009 (Fig 7). Regarding the infection of nymphs by *B*. *afzelii*, a significant difference was found between the forests (p <0.00001). No difference was observed between Sénart (47.3%) and Notre-Dame (37.5%) but a difference was found between Sénart and Rambouillet (11.9%) (p<0.00001) and between Notre-Dame and Rambouillet (p<0.0009). Similarly, there is a significant difference in the infection of nymphs with *B*. *garinii* between the different forests (p <0.0001) (Fig 7). Indeed, this is the species mainly found in the nymphs of Rambouillet forest (41.3% of all species) while the levels are significantly lower for nymphs of Sénart (11.4%) (p<0.00001). There is no significant difference in the infection of nymphs with *B*. *burgdorferi* ss between the different forests. There is a significant difference for *B*.*valaisiana*, in Rambouillet where the prevalence of this species is higher (16.5%) than in Sénart (4.8%) (p<0.0011). Regarding *B*. *spielmanii* and *B*. *lusitaniae*, and co-infections, there is no significant difference in the infection of ticks between the various forests (Fig 7).

#### *Borrelia* species found in Siberian chipmunks captured in the Sénart forest

Eighty-one chipmunks were captured in September 2008. We extracted 81 ear samples that were analyzed by PCR and RFLP. Sixty three percent of the 81 ear samples were found *Borrelia*-positive by PCR. Of the 60 ear samples cultured, 23 (38.3%) were positive for *Borrelia burgdorferii* sl. and eighteen of these were grown (30%).

**Fig 8:**
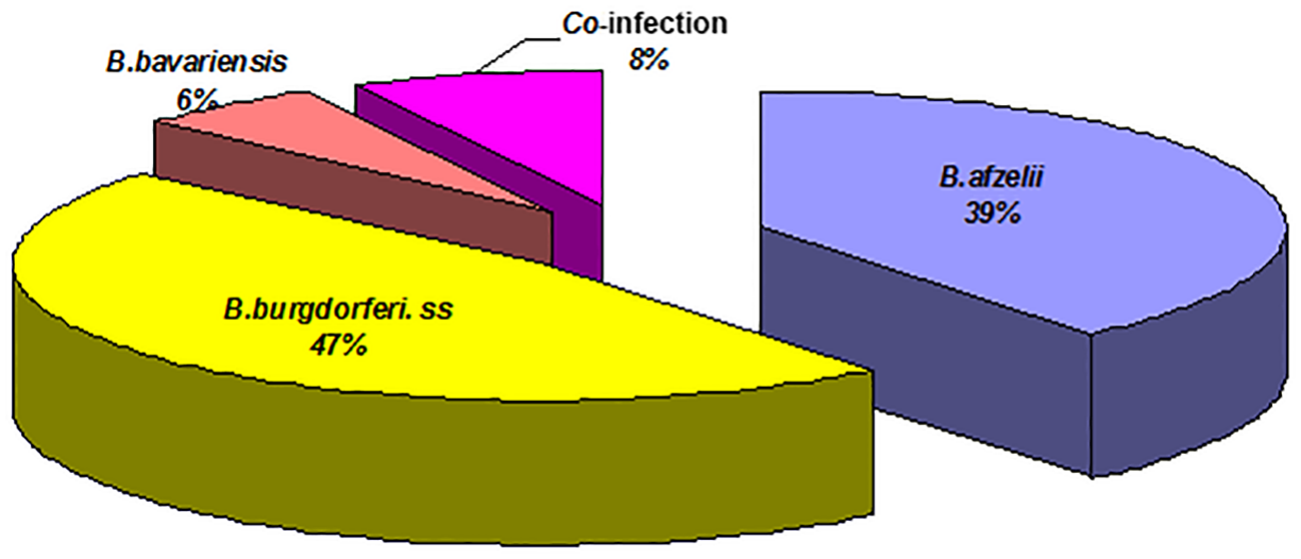
Infection rates (%) of *Borrelia* species identified in Siberian chipmunks collected in Sénart in 2008.

Siberian chipmunks are infected with only three species of *Borrelia* (*B*. *afzelii*, *B*. *burgdorferi* ss and *B*. *bavariensis*) as shown in Fig 8.

#### Tree species

We first considered the correlation between the density of nymphs and adults with the tree species and focused our analysis on plots of Sénart forest. According to correlation tests between the nymph and adult density per plot and vegetation of these plots, only three tree species gave significant p values: chestnut (p<0.00001), ash (p<0.01) and pines (p<0.001). However, only the chestnut tree has a good correlation coefficient for 2008 and 2011 (R > 0.6) (Table 5). Similarly for adult ticks, only the correlation coefficient between adult density and the percentage of chestnut tree was significant (R>0.6) (Table 5). We observe only a moderate correlation during 2009 both for nymph and adult densities (Table 5). We thus observed a positive correlation between the density of *I*. *ricinus* and the percentage of chestnut trees in the plots examined during 2008 and 2011, density which was high in NC plots of the Sénart forest. The other characteristics (rate of infection, density of infected nymphs and adults) did not vary according to the tree species.

**Table 5:** Correlation between the presence of chestnut trees and the tick density.

	Nymphs density		Adult density	
	R	p_value	R	p_value
**2011**	0.74	7.4e^-12^	0.74	1.475e^-11^
**2009**	0.35	0.005	0.45	0.0002
**2008**	0.64	2.7e^-07^	0.61	1.189e^-07^
**3 years**	0.57	2.2e^-16^	0.59	2.2e^-16^
**2011+2008**	0.67	2.2e^-16^	0.68	2.2e^-16^

Table 6 summarizes the influence of several factors (environmental, animal, year) on tick density and infection.

**Table 6:** Summary of all factors that influence tick density and infection.

Factor	Density of Nymphs	Density of infected Nymphs	Density of Adults	Density of infected Adults	Infection rate of Nymphs	Infection rate of Adults
**Year**	No	Yes	No	No	Yes	No
		2009>2008			2009>2008 = 2011	
**Chipmunks (SNC/SC plots)**	Yes	Yes	Yes	Yes	Yes	Yes
	SNC>SC	SNC>SC	SNC>SC	SNC>SC	SC>SNC	SC>SNC
	2008, 2009, 2011	2011	2008, 2009, 2011	2008 and 2011	2008, 2009, 2011	2009
Chipmunks* **(α/β/γ plots)**	Yes	Yes	Yes	Yes	Yes	Yes
	γ = β> α	γ = β > α	γ > β > α	γ = β > α	α > β = γ	α = β > γ
**Chestnut tree**	Yes	No	Yes	No	No	No
**Forest: Sénart Rambouillet Notre-Dame**	No	Yes	No	No	Yes	No
		S>R			S>R	
**Forest: Sénart NC RambouilletNotre-Dame**	No	Yes	Yes	No	No	Yes
		SNC>R	SNC >R = ND			R>NC

* We combined the results obtained during the three years of collect.

S: Sénart forest; R: Rambouillet forest

#### Phylogenetic analyses of *Borrelia* amplification products of rrf-rrl spacer

**Fig 9:**
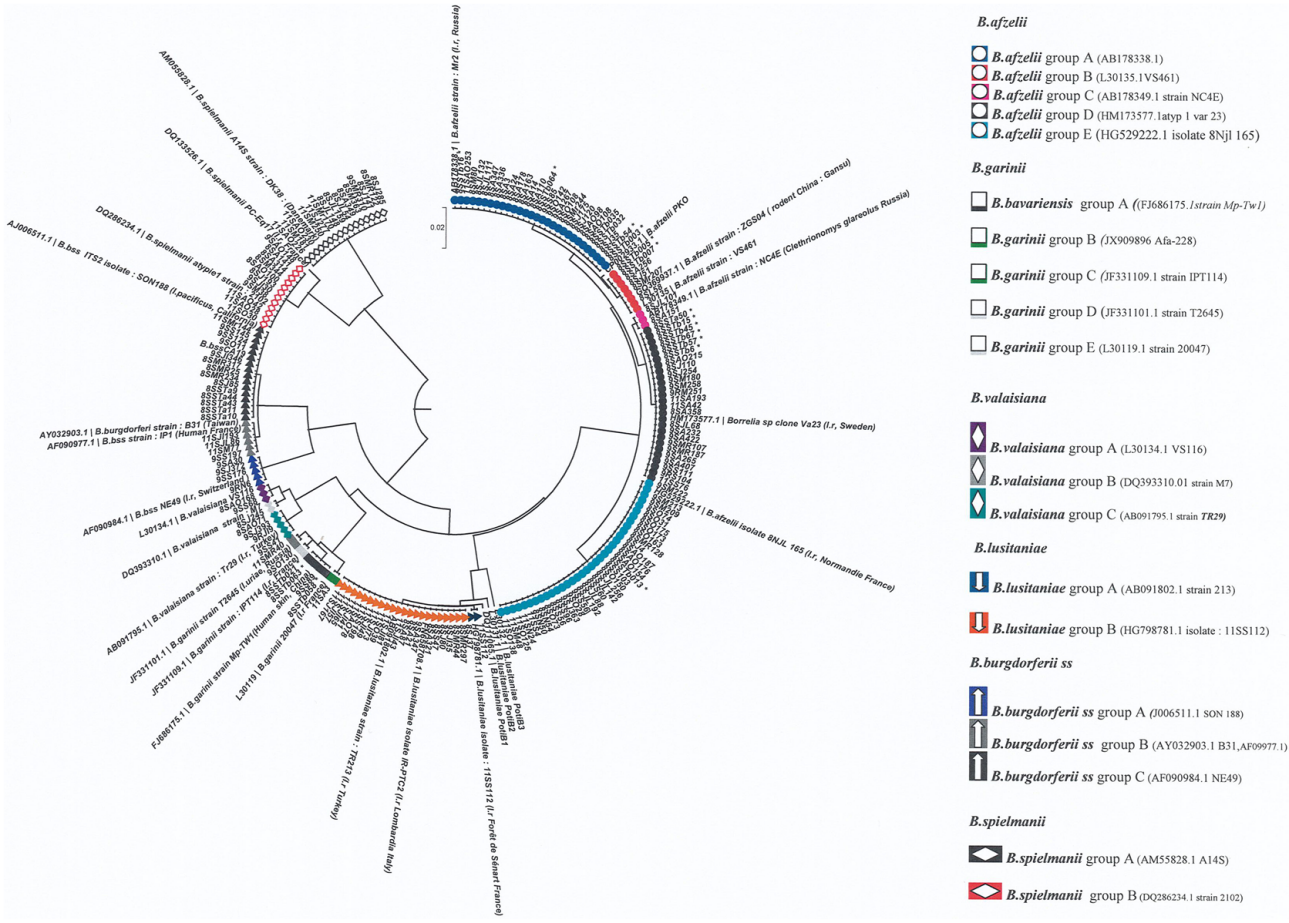
Phylogenetic tree drawn from the sequences of the amplification products from the rrf-rrl space from ticks and chipmunks (T) collected in 2008, 2009 and 2011. The software used for drawing the tree was MEGA 5 (UPGMA method).

The patterns obtained after digestion of the *rrf-rrl* spacer with *Mse*1 allowed various strains belonging to various groups in several species to be identified (Table 7) (Fig 9). Several cases of co-infections were detected, the more frequent implying *B*. *afzelii* and *B*. *burgdorferi* ss on one hand and *B*. *garinii* and *B*. *valaisiana*, on the other hand. To further evaluate the polymorphism, the spacer regions of the strains whose *Mse*1restriction profiles somewhat differ from that of reference strains were sequenced and compared with sequences available in databases. The phylogenetic analyses of *rrf-rrl* spacer sequences were conducted by neighbour joining.

**Table 7:** Polymorphism of *Borrelia burgdorferi* sensu lato from *Mse*I restriction profiles.

*Borrelia* species	restriction profile of *Mse*I (bp)	Group	Sénart 2008		Sénart 2009		Sénart 2009		Notre-Dame 2009		Rambouillet 2009		Chipmunk 2008		Total	
			number	sequence number	number	sequence number	number	sequence number	number	sequence number	number	sequence number	number	sequence number	number	sequence number
*B*. *afzelii* (n = 274)	107, 68, 51, 20	A (AB178338.1)	14	14	3	3	1	1					6	6	24	24
	107, 68, 51, 20	B (VS461^T^) (L30135.1)	18	4	83		27		14		26		7	2	175	6
	107, 68, 51, 20	C (AB178149.1)	2	2											2	2
	107, 68, 51, 13, 7	D (HM173577.1)	3	3	10	10	3	3			2	2	6	6	24	24
	107, 51, 38, 30, 20	E (HG529222.1)	22	22	20	6	6	5					1	1	49	34
*B*. *burgdorferi* ss(n = 261)	107, 52, 40, 29, 28	A (AF497979.1)	29	4	25	3	15	1	7	1	8		11	5	97	14
	107, 52, 38, 29, 28	B31^T^groupB (AF090977.1)	33		50	1	34	3	7		26		13		161	4
	107, 53, 38, 29, 26	C (NE49) (AF090984.1)			2	2					1	1			3	3
*B*. *garinii* (n = 190)	107, 95, 51	A(FJ686175.*1strain Mp-Tw1)*			1	1							1	1	2	2
	107, 95, 51	B (JX909896 Afa-228)											2	2	2	2
	107, 95, 51	C (JF331109.1 IPT 114)			1	1									1	1
	107, 95, 51	D (JF331101.1 T2645)					1	1							1	1
	107, 95, 51	E type 20047^T^ (L30119.1)	48		35		38	1	8		60				189	1
*B*. *lusitaniae* (n = 21)	107 81 39 29	A (TR213) (AB091802.1)	6	6	1	1	12	11							19	18
	107 81 39 29	B (HG798781.1 isolate: 11SS112)	1	1			1	1							2	2
*B*. *spielmanii* (n = 19)	107, 99	PC-EQ17NS^T^ (DQ133526.1))														
	107, 65, 51	A (A14S) (AM055828.1)	3	3	2	1	6	6	1	1					12	11
	107, 67, 48	B (DQ286234.1)	1	1	2	2	4	4							7	7
*B*. *valaisiana* (n = 103)	174, 51, 23, 7	A VS116^T^ (L30134.1)	28	1	16	1	21		4		24				93	2
	204, 51	B (DQ393310.1)	1	1	1		1	1			1				4	2
	174, 51,14, 7	C (AB091795.1)	2	1			1		2	2	1	1			6	4
*B*. *afzelii+B*. *burgdorferi* ss			3		8		4		2		4		1		22	
*B*. *afzelii+B*.*burgdorferi* ss CA 19			1				1						3		5	
*B*. *afzelii+B*. *garinii*											1				1	
*B*. *afzelii atype1+B*. *burgdorferi* ss					2		1								3	
*B*. *afzelii+B*. *valaisiana*							1								1	
*B*. *garinii+B*. *valaisiana*			1		3		1				5				10	
*B*. *garinii+B*. *burgdorferi ss* CA 19					1										1	
*B*. *garinii+B*. *burgdorferi ss*					1										1	
*B*. *burgdorferi ss +B*. *burgdorferi* ss CA19			1												1	

Sequence analysis of *B*. *afzelii* identified five subtypes/groups consistent with the existence of various restriction patterns, in 2008, 2009 and 2011 (Fig 9, Table 7, S4 Fig). Group A contains sequences identical to AB178338.8 isolated from *I*. *ricinus* in Russia [32] and Group B contains sequences identical to GQ369937 isolated from rodents in China [33]. Group C contains sequences identical to AB178349 isolated from rodents in Russia [32] while group D contains sequences identical to HM173577 isolated from *I*. *ricinus* in Sweden [34]. We did not find any sequences in the databank 100% identical to that of group E. This group was new and identified in ticks as well as in *Tamias sibiricus* collected in the Sénart forest, and also in ticks collected in Normandy, another region of North-west France. This strain was introduced in the databank under the number HG529222.1.

*B*. *burgdorferi* ss strains isolated belong to the three groups (A, B and C) previously identified: *B*. *burgdorferi* ss genotype A26 (AF497979.1) *(I*. *ricinus*, Czech Republic) [35], *B*. *burgdorferi* ss B31 (AF0900984.1) [36], and *B*. *burgdorferi* NE49 (AF090984.1) [37] (S5 Fig). Interestingly, group B contains two *B*. *burgdorferi* ss isolates from human cerebrospinal fluid (CSF): AF090977.1 [37] and KY594010, which were found identical to tick sequences of Sénart.

Sequences of *B*. *garinii* were clustered in five groups (A, B, C, D and E), all corresponding to sequences isolated in *Ixodes* ticks except those of group A. Sequences of group A were identical to PBi, a *B*. *bavariensis* sequence type (FJ686175.1) [38] that was identified in chipmunks and ticks as well as in human skin in China [39]. Group B contains sequences isolated from *Tamias sibiricus* of Sénart and was also identified in *Ixodes ricinus* ticks of Finland (JX909896.1) [40] and Germany (Z77176.1). Group C corresponds to the *B*. *garinii* sequence type IPT114 (JF331109) [41]. Group D contains *B*.*garinii* sequences identical to JF331101.1 isolated in *Ixodes uriae* in Russia [41] while Group E contains sequences identical to the *B*. *garinii* sequence type 20047 (L30119) (Fig 9, S6 Fig) [30].

Sequences of *B*. *lusitaniae* are grouped in two clusters. Sequences of group A are grouped in a cluster with strains isolated in Turkey and Italy (AB091802.1) (S7 Fig, Fig 9) [42]. In 2008 and 2011, a new strain was identified (HG798781.1), forming a new cluster of sequences. Group A and B are in a different cluster to the PotiB1, B2 and B3 type strains.

Sequences of *B*. *valaisiana* were grouped in three clusters (S8 Fig). Group A contains sequences identical to L30134 [30], group B contains sequences identical to DQ393310.1 [43] and group C is identical to AB091795.1 isolated from *I*. *ricinus* in Turkey [42].

Concerning *B*.*spielmanii* sequences, none was found identical to *B*. *spielmanii* DQ133 526.1 [31]. Those found were either *B*. *spielmanii* A14S from Denmark or *B*. *spielmanii* DQ286234.1. (S9 Fig) [31].

Analysis of *B*. *afzelii* sequences identified in Siberian chipmunks showed that they were clustered in four groups (A, B, D, E). We did not find any sequence identical to the PKO strain [44] or sequences matching the group C defined for tick sequences. *B*. *burgdorferi* ss strains isolated from *Tamias sibiricus* belonged to Group A and B whereas sequences identical to *B*. *bavariensis* PBi were also identified (S10 Fig).

## Discussion

Lyme disease is a zoonotic disease that poses an important public health problem in urbanized areas where increasingly large numbers of people attend urban forests and park settings. Understanding parameters of human risk of exposure to Lyme disease is critical to target prevention, control, and surveillance actions. In this purpose, the present study was undertaken to examine the spatial and temporal dynamics of *I*. *ricinus* in three peri-urban forests of the Île-de-France region and to establish their relationship to *Borrelia* infection rates of ticks collected at selected study sites. Indeed, although the ecology of Lyme borreliosis is reasonably well understood, the factors that determine the observed large variations in tick density, infection rates, and other determinants for Lyme borreliosis are not.

The Sénart forest is a 3200 ha suburban forest 22 km southeast of Paris, France. This forest was especially studied to determine the effect of the introduction of a new host species on vector dynamics. This rodent, whose original distribution range extends from eastern Finland to the Bering Strait and East Asia, has quickly adapted to its new environment in Europe. One of the largest populations is located in the Sénart forest and accounts for between 10,000 and 20,000 individuals. There is field evidence that *T*. *sibiricus* can be infected by *Borrelia* sp. including both *B*. *burgdorferi* ss and *B*. *afzelii* [45–50]. Moreover, Siberian chipmunks are suspected to contribute to Lyme borreliosis risk, as they host higher numbers ticks and are more often infected by diverse *Borrelia* genospecies than the native rodent reservoir species. However, recent studies have shown that although they are infected with *Borrelia* species, they do not seem to be persistently infected [51].

Considering these various results, we decided to investigate their putative influence on the density of ticks, on their infection rate and their density of infection overtime. Ticks were collected on eight plots in the forest of Sénart. Five plots were in the area colonized by Siberian chipmunks (between 2 and 5 individuals per ha) (western part of the forest), one located in an intermediate area recently colonized where few animals are settled (north-east) and 2 in the south-east, an area without chipmunk. In order to detect a spatial variation, 8 and 2 plots were also collected respectively in the forests of Rambouillet and Notre-Dame, two forests that are considered "without chipmunk." The study was performed during several months over three years to detect seasonal, temporal and spatial variations.

### Spatio-temporal and seasonal variations in tick abundance and infection

There was a seasonal variation in questing nymph abundance, with high densities in spring and summer in the Sénart forest in the three years of collect. Similar seasonal patterns were observed in the Notre-Dame and Rambouillet forests. Considerable monthly variations in *I*. *ricinus* density were also reported in two other French departments: Meuse and the Puy-de-Dôme [7]. The reasons for such differences are probably explained by diverse competing environmental factors: mainly climatic conditions [52], vegetation type, and the abundance of wild hosts [53]. Such a distribution has been previously observed in British woodland [54].

Temporal variations were also studied in the Sénart forest. In terms of the density of nymphs and adult ticks, no difference was recorded between the three years of collection. In contrast, the rate of infection of nymphs was higher in 2009 than in 2008 and 2011. Ostfeld et al. [12] conclude that interannual variation in entomological risk of exposure to Lyme disease is correlated positively with prior abundance of key hosts for the immature stages of tick vectors. Captures of rodents were performed in 2006, 2007 and 2008 in the Sénart forest. They show that the number of rodent captured was higher in 2008 than during the preceding years. This information can explain the higher rate of infection and density of infected nymphs in 2009. The density of infected adults remained similar during the three years of study whereas that of nymphs was higher in 2009.

Spatial variations were also studied between the different forests studied. The density of questing nymphs and adults was similar. The rate of infection of nymphs was higher in Sénart compared to Rambouillet. In agreement, the density of infected nymphs was higher in Sénart compared to Rambouillet (respectively 12 per 100 m^2^ and 5 per/100 m^2^). These results are similar to the densities of infected ticks observed in 2 other departments of France: Meuse and the Puy-de-Dôme, but inferior to that found in Alsace [6, 7].

### Prevalence of *Borrelia* species in the three forests

A high diversity of species was found in the three forests since six genospecies of *B*. *burgdorferi* sl were present in the collected *I*. *ricinus* ticks. Several cases of co-infections were identified. The most frequent associated *B*. *burgdorferi* ss and *B*. *afzelii* on one hand and *B*. *garinii* and *B*. *valaisiana* on the other hand. This observation is in good correlation with the reservoirs that are shared between these genospecies. Hence, *B*. *garinii* and *B*. *valaisiana* are more often associated with birds [15, 55, 56], whereas small rodents are reservoir hosts for *B*. *afzelii* and *B*. *burgdorferi* ss [16, 57–59]. *B*. *burgdorferi* ss is found in both rodents and birds. We also found *B*. *lusitaniae*, a species rarely found in Northern Europe. *B*. *lusitaniae* is mainly driven by lizards [60] and birds [61]. They were mostly identified in ticks collected in the SE3 plot emphasizing the observation that this strain is focally distributed in central and Eastern Europe.

It is interesting that most *B*. *lusitaniae* found in Sénart were 100% identical with strains found in Italy and in Turkey [34]. It is possible that this *Borrelia* species, rare in northern Europe, was disseminated by the same migratory birds. Indeed, among the bird species on which infected ticks by *B*.*lusitaniae* were found, we distinguish *Turdus philomelos* [61]. This bird migrates from the Nordic countries to France in the summer and overwinters in Turkey and North Africa [62]. The role of this bird in the dispersal of *B*. *lusitaniae* has been highly suspected. A new variant of *B*. *lusitaniae* was identified in our study.

We observed geographical differences in the various *Borrelia* species prevalences in the various forests. The prevalence of *B*. *afzelii* was low in the nymphs of the Rambouillet forest whereas that of *B*. *garinii* was high. Also, the frequency of *B*. *valaisiana* was higher in Rambouillet than in the other forests. These local disparities may be related to factors such as the presence/density of reservoir hosts, forest structure, and types of biotope. Indeed, variation in animal host species composition is an important factor in determining tick-associated bacterial communities [14]. Our results suggest that the reservoir host composition of Rambouillet may be unique and may contain a high diversity in the bird community. *Borrelia* genospecies prevalence found in Rambouillet was similar to the *Borrelia* diversity identified in ticks captured in Germany where *B*. *garinii* was the most frequently detected species [63]. It is well-known that the prevalence of *B*. *garinii* increases from Western to Eastern Europe [64] and this gradient may explain the observations made in Germany. However, our results differ from those found in the Netherlands, Denmark and Belarus [65–67], where *B*. *afzelii* was predominant among *B*. *burgdorferi* sl species. Since *B*. *garinii* is associated with neuroborreliosis [68], our findings may suggest a higher risk to develop this form of Lyme borreliosis after a tick bite in Rambouillet. However, we found *B*. *burgdorferi* ss rrf-rrl spacer sequences that were identical to those found in human CSF collected in France emphasising on the role this genospecies plays in neurological symptoms associated to Lyme disease.

We also found a temporal variation during the three years of collect in the prevalence of the *B*. *afzelii* both in nymph and adults of *I*. *ricinus* ticks in the Sénart forest, which was higher in 2009 compared to the other years. *B*. *afzelii* is mostly associated with rodents and the population of these animals was shown to vary according to the year.

### Role of *Tamias sibiricus* in the Sénart forest

The Sénart forest is the only one among the three forests where Siberian chipmunks were introduced. Recent work in this forest showed a rate of parasitism by *Ixodes ricinus* and infection rates by *B*. *burgdorferi*. ss, *B*. *afzelii* and *B*. *garinii* greater in chipmunks than in other indigenous rodent species tested, namely the bank vole and the wood mouse [49, 69]. In the present study, the presence of chipmunks was shown to be correlated to both the density and the rate of infection of both nymphs and adults. A higher density of nymphs and adults was observed in NC plots (south-eastern part of the forest), and conversely a higher rate of infection of nymphs was obtained in C plots (western part of the forest) in 2008, 2009 and 2011. Interestingly, the plot being colonized recently (north-eastern part of the forest) has both a rate of infection of nymph and density of infected nymph and adult similar to non-colonized plots whereas the density of adult is intermediate between colonized and non-colonized plots. The density of infected nymphs is known to characterize the acarological risk. We observe only in 2011 a higher density of infected nymphs in the south-eastern part of the forest, as shown by Vourc’h et al. during one month of the same year [70], whereas no difference was reported in 2008 and 2009, showing the variation of this important parameter over year in this particular forest.

### Factors that may influence tick density and infection

#### Tree species

Vegetation plays an important role in the tick life-cycle. Diuk-Wasser et al. [71] reported that the abundance of *I*. *scapularis* appeared associated with specific sites, having different host abundances. In Europe, Jouda et al. [72] reported similar associations between sites and ticks. In particular, thickness of litter layer and cover of the moss layer were positively correlated with tick density. Vourc’h et al. [70] found a positive correlation between deciduous trees (mainly oaks) and the density of nymphs in the Sénart forest in May 2011. Interestingly, our results show that only the number of chestnut trees correlated positively with nymph and adult density in 2008 and 2011 consistent with the results of Ceballos et al., [73]. Chestnut trees are valued for their leaves, flowers and mainly for fruit by wild boars, roe deer and rodents. Therefore, its presence increases the food supplies in plots that are rich in chestnut trees. We can put forward the hypothesis that the chestnuts presence attracts these mammals, which will spend more time in this vegetation. In addition to food, chestnut stump sprouts provide denser undergrowth (as coppiced wood) than other species (except hornbeam or common hazel). This offers a better shelter in the day towards walkers than the undergrowth of dominant oaks. Ticks at the end of a blood meal on these hosts will then have a greater probability of falling below the fruit trees, which could explain their high density in these plots. Interestingly, other studies have demonstrated a link between the tick density and the nature of vegetation [74].

#### Host composition

Moreover, apart from habitat characteristics, host composition and abundance may affect tick population [75–77]. In a number of tick-pathogen systems, certain tick hosts do not support the multiplication of the pathogen. Incompetent hosts may play a crucial role in determining the infection prevalence in the vectors [78]. It has been proposed for natural communities that the abundance of inefficient hosts for the transmission of the pathogen to a feeding vector could act as a diluting factor in the dynamics of pathogen transmission, thereby reducing the exposure rate in competent hosts. In the case of Lyme disease, deer are refractory to infection but feed a large number of ticks. Our results therefore suggest that the abundance of roe deer may be higher in plots of the south-eastern part (NC plots) than in those of the western part (C plots), and that their presence may be related to particular habitat characteristics of this part of the Sénart forest. In correlation with our results, the ONF showed in 2008 that the population of roe deer and wild boars were higher in the NC plots than in C plots. Conversely, the high rate of infection in C plots may be explained by the presence of a higher abundance of reservoir hosts, among which chipmunks that are particularly susceptible to infection by *Borrelia burgdorferi* sl.

### Conclusions

In conclusion, our study provides interesting insights concerning the spatio-temporal distribution as well as the infection of ticks collected in peri-urban forests, which are important sites of recreation for people. We observed that the density of nymphs and adults was stable overtime in the Sénart forest but some parameters like the infected nymph density may vary according to the year of tick collection. Concerning the putative role that *Tamias sibiricus* may play in the transmission of *Borrelia* in the Sénart forest, our results suggest that this newly introduced species may be involved in the rate of infection of ticks by *Borrelia* genospecies and if its population increases, it could play a significant role in *Borrelia* transmission. The acarological risk in the sampled plots located respectively in western and eastern parts of the Sénart forest, was found however similar in 2008 and 2009, and higher to that recorded in other forests of the same region. We think that it is important not to draw attention to any particular part of the Sénart forest or to any particular forest for prevention and control measures against *Borrelia burgdorferi* sl transmission but rather to inform people that a risk exists in any forest. However, we must stress that the risk identified in peri-urban forests of Île-de-France is inferior to what was found in Alsace where the rate of infestation of ticks by *Borrelia* and the density of infected nymphs were significantly higher [6].

## Supporting information

S1 Table Localization and description of the various plots where ticks were collected. (DOC)

S2 Table Density, infection rate and density of infected ticks of *I*. *ricinus* in the forest of Sénart in 2008, 2009 and 2011 on eight plots. (DOC)

S3 Table Correlations across active variables used for principal component analysis of the Sénart forest. (DOC)

S4 Table Density and infection status of ticks *I*. *ricinus* in the Notre-Dame, Rambouillet and Sénart forest in 2009. (DOC)

S5 Table Density and infection status of ticks *I*. *ricinus* in α, β and γ plots of the forest of Sénart in 2008, 2009 and 2011. (DOC)

S6 Table Prevalence of *Borrelia* species identified in NC and C plots of Sénart forest. (DOCX)

S7 Table Prevalence of *Borrelia* species in adult collected in 2008, 2009 and 2011 in α, β and γ plots of the Sénart forest. (DOC)

S1 Fig Scatter plot of observations in the factorial map according to the year of the experiment. The dots are colored according year of the experiment.
(TIF)

S2 Fig Scatter plot of observations in the factorial map according to the month of the experiment. The dots are colored according to the month of the experiment.
(TIF)

S3 Fig Scatter plot of observations in the factorial map according to chipmunk abundance. The dots are colored according to chipmunk abundance.
(TIF)

S4 Fig Phylogenetic tree obtained from the DNA sequences of the rrf-rrl amplicons of *B*. *afzelii*. The software used for drawing the tree was MEGA 5 (UPGMA method).
(DOC)

S5 Fig Phylogenetic tree obtained from the DNA sequences of the rrf-rrl amplicons of *B*. *burgdorferi* ss. The software used for drawing the tree was MEGA 5 (UPGMA method).
(DOC)

S6 Fig Phylogenetic tree obtained from the DNA sequences of the rrf-rrl amplicons of *B*. *garinii* and *B*. *bavariensis*. The software used for drawing the tree was MEGA 5 (UPGMA method).
(DOC)

S7 Fig Phylogenetic tree obtained from the DNA sequences of the rrf-rrl amplicons of *B*. *lusitaniae*. The software used for drawing the tree was MEGA 5 (UPGMA method).
(DOC)

S8 Fig Phylogenetic tree obtained from the DNA sequences of the rrf-rrl amplicons of *B*. *valaisiana*. The software used for drawing the tree was MEGA 5 (UPGMA method).
(DOC)

S9 Fig Phylogenetic tree obtained from the DNA sequences of the rrf-rrl amplicons of *B*. *spielmanii*. The software used for drawing the tree was MEGA 5 (UPGMA method).
(DOC)

S10 Fig Phylogenetic tree obtained from the DNA sequences of the rrf-rrl amplicons of *Borrelia* genospecies in *Tamias sibiricus*. The software used for drawing the tree was MEGA 5 (UPGMA method).
(DOC)
