# Supplementary material for: Infection of Ixodes ricinus by Borrelia burgdorferi sensu lato in peri-urban forests of France
Source: PLoS One. 2017 Aug 28;12(8):e0183543. doi: 10.1371/journal.pone.0183543 (PMC5573218; doi:10.1371/journal.pone.0183543)
Supplement: S1 Table — (DOC) [file pone.0183543.s001.doc]

Supplementary table 1

| Forest Sénart  2008-2009 | plot SE1 | plot SE2 | plot SE3 | plot SE4 | plot SE5 | plot SE6 | plot SE8 | plot SE9 |
| --- | --- | --- | --- | --- | --- | --- | --- | --- |
| time zone | 31 | 31 | 31 | 31 | 31 | 31 | 31 | 31 |
| zone | U | U | U | U | U | U | U | U |
| longitude | 463377 | 461632 | 459165 | 459940 | 458132 | 462507 | 465254 | 465823 |
| latitude | 5392275 | 5391676 | 5390141 | 5390053 | 5391511 | 5388351 | 5389755 | 5389520 |
| altitude (m) | 88 | 85 | 62 | 86 | 155 | 46 | 88 | 84 |
| Vegetation |  |  |  |  |  |  |  |  |
| Predominant species 1 | chestnut | pines | oaks | oaks | oaks | oaks | chestnut | chestnut |
| Predominant species 2 | oaks | oaks | birches | hornbeams | chestnut | hornbeams | oaks | oaks |
| Others | hornbeams | hornbeams | hornbeams | lime | beech | chestnut | hornbeams | hornbeams |
|  | brambles | brambles | chestnut | chestnut | brambles | brambles | brambles | brambles |
|  | anemones | ferns | brambles | brambles |  |  | anemones | anemones |
|  |  |  | ferns | thrush |  |  |  |  |
|  |  |  |  | ferns |  |  |  |  |
| dwelling | near | far | far | far | far | far | far | far |

| Forest Rambouillet  2009 | plot  R2 | plot  R5 | plot  R6 | plot  R7 | plot  R8 | plot  R10 | plot  R17 | plot  R20 |
| --- | --- | --- | --- | --- | --- | --- | --- | --- |
| time zone | 31 | 31 | 31 | 31 | 31 | 31 | 31 | 31 |
| Zone | U | U | U | U | U | U | U | U |
| Longitude | 0418052 | 417721 | 0414784 | 0405688 | 0405651 | 04060097 | 411600 | 412500 |
| Latitude | 5388114 | 5886530 | 5386591 | 5392128 | 5392097 | 541861 | 5400575 | 5398325 |
| Altitude (m) | 193 | 177 | 155 | 127 | 277 | 79 | 134 |  |
| Vegetation |  |  |  |  |  |  |  |  |
| Predominant species 1 | oaks | oaks | oaks | oaks | oaks | oaks | pines | beech |
| Predominant species2 | Moor grass | pines | pines | hornbeams | hornbeams | birch | ferns | firs |
| Others |  | beech |  |  |  | hornbeams | brambles | birch |
|  |  |  |  |  |  | ferns | regeneration | brambles  regeneration |
| dwelling | far | far | far | far | far | far | far | far |

| Forest  Notre-Dame  2009 | plot  SE10 | plot  SE11 |
| --- | --- | --- |
| time zone | 31 | 31 |
| Zone | U | U |
| Longitude | 0461563 | 0463400 |
| Latitude | 5391648 | 5392770 |
| Altitude (m) | 75 | 88 |
| vegetation | hornbeams | hornbeams |
|  | chestnut | chestnut |
|  | brambles | pines |
| dwelling | near | near |
