# Supplementary material for: Infection of Ixodes ricinus by Borrelia burgdorferi sensu lato in peri-urban forests of France
Source: PLoS One. 2017 Aug 28;12(8):e0183543. doi: 10.1371/journal.pone.0183543 (PMC5573218; doi:10.1371/journal.pone.0183543)
Supplement: S2 Table — (DOC) [file pone.0183543.s002.doc]

Supplementary table 2: Density, infection rate and density of infected ticks of *I. ricinus* in the forest of Sénart in 2008, 2009 and 2011 on eight plots

|  |  |  |  | **2008** |  |  |  |  |  |  |  |  |  | **2009** |  |  |  |  |  |  |  |  |  | **2011** |  |  |  |  |
| --- | --- | --- | --- | --- | --- | --- | --- | --- | --- | --- | --- | --- | --- | --- | --- | --- | --- | --- | --- | --- | --- | --- | --- | --- | --- | --- | --- | --- |
|  | **March** | **April** | **May** | **June** | **July** | **August** | **September** | **October** | **Total** | **March** | **April** | **May** | **June** | **July** | **August** | **September** | **October** | **Total** | **March** | **April** | **May** | **June** | **July** | **August** | **September** | **October** | **Total** | **Stat**  **P** |
| **Nymphs (N)**  Density/100 m^2^  CI | 680  53.1  28-78 | 1148  89.7  43-136 | 1704  133.1  68-198 | 1224  95.6  43-149 | 1147  89.6  20-159 | 617  48.2  11-85 | 422  32.9  9-57 | 220  17.2  6-29 | 7162  69.9 | 1036  80.9  32-130 | 1415  110.6  68-153 | 1350  105.5  60-151 | 2024  158.1  91-226 | 1676  130.9  55-207 | 727  56.8  17-96 | 311  24.3  8-41 | 162  14.5  8-21 | 8701  85.2 | 986  102.7  37-169 | 1594  124.5  50-199 | 2126  166.1  41-291 | 3136  245  67-423 | 1922  150.2  45-255 | 1512  118.1  27-209 | 914  71.4  13-130 | 476  37.2  6.5-68 | 12666  126.9 | NS |
| **Adults** **(N)**  Density/100 m^2^  CI | 35  2.7  2-4 | 72  5.6  0-11 | 61  4.8  1-8 | 90  7  2-12 | 57  4.5  0.6-8 | 30  2.3  0.3-4 | 33  2.6  0.8-4 | 29  2.3  0-4 | 407    4 | 56  4.4  1-8 | 90  7.0  3-11 | 84  6.6  3-10 | 91  7.1  3-11 | 69  5.4  2-9 | 42  3.3  1-6 | 40  3.1  1-5 | 23  2.1  1-4 | 495  4.9 | 30  3.1  0.7-5.5 | 41  3.2  0.3-6 | 89  7  0.8-13.2 | 98  7.7  1.5-13.8 | 34  2.7  0.6-4.7 | 44  3.4  0.3-6.6 | 28  2.2  0.2-6.2 | 36  2.8  0-6 | 400  4 | NS |
| **Nymphs**  Infection rates  % | 27/234  11.5 | 26/236  11 | 27/240  11.3 | 19/257  7.4 | 19/195  9.7 | 12/180  6.7 | 15/161  9.3 | 20/147  13.6 | (1165/1650  10 | 26/225  11.6 | 32/240  13 | 37/240  15 | 32/240  13.3 | 32/239  13.4 | 22/201  10.9 | 21/152  13.8 | 13/145  9 | 215/1682  12.8 | 9/176  5.1 | 11/240  4.6 | 20/221  9.0 | 11/240  4.6 | 32/230  13.9 | 26/220  11.8 | 17/176  9.7 | 13/138  9.4 | 139/1641  8.5 | <0.0002  2009>2008  2008=2011  2009>2011 |
| **Adults**  Infection rates  % | 7/36  19.4 | 4/70  5.71 | 3/61  4.92 | 7/80  8.75 | 7/52  13.5 | 4/29  13.8 | 1/33  3 | 4/29  13.8 | 37/390  9.5 | 1/54  19 | 8/84  9.50 | 11/83  13 | 10/82  13 | 9/67  13 | 9/42  21 | 3/28  8 | 1/23  4 | 52/473  11 | 1/28  3.6 | 3/39  7.7 | 8/73  11.0 | 9/82  11.0 | 3/34  8.8 | 6/44  13.6 | 2/28  7.1 | 2/35  5.7 | 34/363  9.4 | NS |
| **Density of infected**  **nymphs**  Density/100 m^2^ | **6.1** | **9.9** | **15** | **7.1** | **8.7** | **3.2** | **3.1** | **2.3** | **7** | **9** | **15** | **16** | **21** | **18** | **6** | **3** | **1** | **11** | **5.3** | **5.7** | **15.0** | **11.2** | **20.9** | **14.0** | **6.9** | **3.5** | **10.7** | **S**  **<0.024**  2009>2008 |
| **Density of**  **infected adults**  Density/100 m^2^ | **0.5** | **0.3** | **0.2** | **0.6** | **0.6** | **0.3** | **0.1** | **0.3** | **0.4** | **0** | **1** | **1** | **1** | **1** | **1** | **0** | **0** | **0.5** | **0.1** | **0.2** | **0.8** | **0.8** | **0.2** | **0.5** | **0.2** | **0.2** | **0.4** | **NS** |

CI : confidence interval
