# Supplementary material for: Infection of Ixodes ricinus by Borrelia burgdorferi sensu lato in peri-urban forests of France
Source: PLoS One. 2017 Aug 28;12(8):e0183543. doi: 10.1371/journal.pone.0183543 (PMC5573218; doi:10.1371/journal.pone.0183543)
Supplement: S3 Table — (DOC) [file pone.0183543.s003.doc]

Supplementary Table 3: Correlations across active variables used for Principal Component Analysis of the Sénart forest

|  | Hour | Temperature | Hygrometry | Oak% | Chestnut% | Pines% | Density of nymphs | Density of adults | Density infected nymphs | Density infected adults | Infection  rate nymphs | Infection rate  adults |
| --- | --- | --- | --- | --- | --- | --- | --- | --- | --- | --- | --- | --- |
| Hour | 1 | 0.41 | -0.61 | -0.24 | 0.06 | 0.18 | -0.03 | -0.11 | 0.07 | -0.07 | 0.08 | -0.03 |
| Temperature | **0.41** | 1 | **-0.27** | **-0.1** | **-0.09** | **0.12** | **0.05** | **-0.04** | **0.19** | **0.09** | **0.2** | **0.07** |
| Hygrometry | -0.61 | -0.27 | 1 | 0.13 | -0.04 | -0.09 | -0.09 | -0.02 | -0.11 | 0.03 | -0.05 | 0.01 |
| Oak% | -0.24 | -0.1 | 0.13 | 1 | -0.05 | -0.91 | 0.18 | 0.11 | 0.02 | 0.04 | -0.18 | 0.06 |
| Chestnut% | 0.06 | -0.09 | -0.04 | -0.05 | 1 | -0.36 | 0.51 | 0.59 | 0.24 | 0.31 | -0.19 | 0.27 |
| Pines% | 0.18 | 0.12 | -0.09 | -0.91 | -0.36 | 1 | -0.39 | -0.34 | -0.12 | -0.17 | 0.25 | -0.17 |
| Density of nymphs | -0.03 | 0.05 | -0.09 | 0.18 | 0.51 | -0.39 | 1 | **0.75** | **0.69** | **0.47** | **-0.05** | **0.37** |
| Density of adults | -0.11 | -0.04 | -0.02 | 0.11 | 0.59 | -0.34 | 0.75 | 1 | **0.49** | **0.62** | **-0.13** | **0.46** |
| Density infected nymphs | 0.07 | 0.19 | -0.11 | 0.02 | 0.24 | -0.12 | 0.69 | 0.49 | 1 | **0.47** | **0.56** | **0.39** |
| Density infected adults | -0.07 | 0.09 | 0.03 | 0.04 | 0.31 | -0.17 | 0.47 | 0.62 | 0.47 | 1 | **0.07** | **0.86** |
| Infection rate nymphs | 0.08 | 0.2 | -0.05 | -0.18 | -0.19 | 0.25 | -0.05 | -0.13 | 0.56 | 0.07 | 1 | **0.16** |
| Infection rate adults | -0.03 | 0.07 | 0.01 | 0.06 | 0.27 | -0.17 | 0.37 | 0.46 | 0.39 | 0.86 | 0.16 | 1 |
