# Supplementary material for: Infection of Ixodes ricinus by Borrelia burgdorferi sensu lato in peri-urban forests of France
Source: PLoS One. 2017 Aug 28;12(8):e0183543. doi: 10.1371/journal.pone.0183543 (PMC5573218; doi:10.1371/journal.pone.0183543)
Supplement: S4 Table — (DOC) [file pone.0183543.s004.doc]

Supplementary Table 4: Density and infection status of ticks *I. ricinus* in the Notre-Dame, Rambouillet and Sénart forest in 2009

| 2009 | **Notre-Dame** | | | | | | | **Rambouillet** | | | | | | | **Sénart** | | | | | | |  |
| --- | --- | --- | --- | --- | --- | --- | --- | --- | --- | --- | --- | --- | --- | --- | --- | --- | --- | --- | --- | --- | --- | --- |
|  | **April** | **May** | **June** | **July** | **September** | **October** | **Total** | **April** | **May** | **June** | **July** | **September** | **October** | **Total** | **April** | **May** | **June** | **July** | **September** | **October** | **Total** | **Stat**  **p** |
| **Nymphs(N)**  Density/100 m^2^  IC | 138  43.1  31-55 | 45  14.1  10-18 | 272  85  80-90 | 286  89.4  57-124 | 159  49.9  26-74 | 41  12.8  12-13 | **941**  **49.1** | 467  41.7  31-53 | 1014  79.2  33-126 | 1309  102.3  41-164 | 957  74.8  30-120 | 529  41.3  13-64 | 311  24.3  12-37 | **4587**  **60.6** | 1415  110.6  68-153 | 1350  105.5  60-151 | 2024  158.1  91-226 | 1676  130.9  55-207 | 311  24.3  8-41 | 162  14.5  8-21 | **6938**  **90.7** | NS |
| **Adults** **(N)**  Density/100 m^2^  IC | 8  2.5  1-4 | 2  0.6  0-2 | 9  2.8  1-5 | 17  5.3  5-6 | 11  3.4  2-5 | 2  0.6  0-2 | **49**  **2.6** | 34  3  2-5 | 75  5.9  2-9 | 56  4.4  2-7 | 46  3.6  1-6 | 26  2  1-4 | 24  1.9  1-3 | **261**  **3.5** | 90  7  3-11 | 84  6.6  3-10 | 91  7.1  3-11 | 69  5.4  2-9 | 40  3.1  1-5 | 23  2.1  0.5-4 | **397**  **5.2** | NS |
| **Nymphs**  Infection rates  % | 6/60  10 | 2/45  4 | 8/60  13.3 | 5/60  8.3 | 5/60  8.3 | 6/41  14.6 | **32/326**  **9.8** | 19/210  9.1 | 14/240  5.8 | 22/201  10.9 | 25/213  11.7 | 20/202  9.9 | 9/168  5.4 | **109/1234**  **8.8** | 32/240  13.3 | 37/240  15.4 | 32/240  13.3 | 32/239  13.4 | 21/2152  13.8 | 13/145  9 | **167/1256**  **13.3** | < 0.002  S>R |
| **Adults**  Infection rates  % | 0/8  0 | 0/2  0 | 1/9  11 | 1/14  7 | 2/11  18 | 0/2  0 | **4/46**  **8.7** | 3/32  9.4 | 13/70  18.4 | 10/48  20.8 | 9/42  21.4 | 6/24  25 | 1/23  4.3 | **42/239**  **17.6** | 8/84  9.5 | 11/83  13.3 | 10/82  12.2 | 9/67  13.4 | 3/38  7.9 | 1/23  4.3 | **42/377**  **11.1** | NS |
| **Density of nymphs**  **infected**  Density/100 m^2^ | **4.3** | **0.6** | **11.3** | **7.4** | **4.1** | **1.9** | **4.8** | **3.8** | **4.6** | **11.2** | **8.8** | **4.1** | **1.3** | **5.3** | **14.7** | **16.3** | **21.1** | **17.5** | **3.4** | **1.3** | **12.1** | **<0.002**  **S>R** |
| **Density of**  **adults**  **infected**  Density/100 m^2^ | **0** | **0** | **0.3** | **0.4** | **0.6** | **0** | **0.2** | **0.3** | **1.1** | **0.9** | **0.8** | **0.5** | **0.1** | **0.7** | **0.7** | **0.9** | **0.9** | **0.7** | **0.2** | **0.1** | **0.6** | **NS** |

Ticks were not collected during March and August at Notre-Dame and Rambouillet forests. We therefore removed these two months in the comparison with Sénart forest.
