# Supplementary material for: Infection of Ixodes ricinus by Borrelia burgdorferi sensu lato in peri-urban forests of France
Source: PLoS One. 2017 Aug 28;12(8):e0183543. doi: 10.1371/journal.pone.0183543 (PMC5573218; doi:10.1371/journal.pone.0183543)
Supplement: S5 Table — (DOC) [file pone.0183543.s005.doc]

Supplementary Table 5: Density and infection status of ticks *I. ricinus* in α, β, γ plots of the forest of Sénart in 2008, 2009 and 2011

|  | **2008**  α **plots** | **2008**  β **plot** | **2008**  γ **plots** | **Stat**  **2008**  γ **/** α **/** β  **plots** | **2009**  α **plots** | **2009**  β **plot** | **2009**  γ **plots** | **Stat**  **2009**  γ **/** α **/** β  **plots** | **2011**  α **plots** | **2011**  β **plot** | **2011**  γ **plots** | **Stat**  **2011**  γ **/** α **/** β  **plots** | **Stat**  **2008-2009-2011**  α **plots** | **Stat**  **2008-2009-2011**  β **plot** | **Stat**  **2008-2009-2011**  γ **plots** | **Stat**  **2008 2009-2011**  **total** |
| --- | --- | --- | --- | --- | --- | --- | --- | --- | --- | --- | --- | --- | --- | --- | --- | --- |
| **Nymphs(N)**  Density/100 m^2^ | 2071  32.4 | 2188  170.9 | 2903  113.4 | S  <0.0001  α **<** β  α **<** γ  γ = β  β = γ > α | 3197  50.2 | 1605  125.4 | 3899  152.3 | S  <0.0002  α **<** γ  α **<** β  γ = β  β = γ > α | 2523  41.1 | 2443  190.9 | 7700  300.8 | S  <0.0001  α **<** γ  α **<** β  γ = β  γ = β > α | NS | NS | S  <0.002  2008=2009  2008<2011  2009<2011  2011>2009=2008 | NS |
| **Adults** **(N)**  Density/100 m^2^ | 94  1.5 | 74  5.8 | 239  9.3 | S  <0.0001  α **<** β  α **<** γ  γ = β  γ = β > α | 151  2.4 | 93  7.3 | 251  9.8 | S  <0.00001  α **<** β  α **<** γ  γ = β  γ = β > α | 62  1 | 60  4.7 | (278)  10.9 | S  <0.00001  α **<** β  α **<** γ  γ > β  γ > β > α | NS | NS | NS | NS |
| **Nymphs**  Infection rates % | 113/930  12.2 | 29/240  12.1 | 23/480  4.8 | S  <0.00004  γ **<** α  γ **<** β  α = β  α = β > γ | 152/962  15.8 | 22/240  9.2 | 41/480  8.5 | S  <0.0001  γ **<** α  β **<** α  β = γ  α > β = γ | 96/919  10.4 | 16/240  6.7 | 27/482  5.6 | S  <0.002  γ **<** α  β **=** α  β **=** γ | S<0.002  **2009>2011**  2009= 2008  200=2011 | NS | NS | **S<0.0002**  **2009>2008**  **2009>2011**  **2008=2011** |
| **Adults**  Infection rates % | 7/87  8 | 11/72  15.2 | 19/151  15.2 | NS | 15/139  10.8% | 19/91  20.9% | 18/243  7.4% | S  <0.002  β = α  γ = α  γ < β | 7/61  11.5 | 5/58  8.6 | 22/244  9 | NS | NS | NS | NS | NS |
| **Density of nymphs**  **infected**  Density/100 m^2^ | 3.9 | 20.7 | 5.4 | S  <0.0003  α < β  γ > β  α = γ  β > γ = α | 7.9 | 11.5 | 13 | NS | 4.3 | 12.7 | 16.8 | S  <0.0003  γ = β  α < γ  α < β  γ = β > α | NS | NS | S  <0.0057  2008<2009  2008<2011  2009=2011  2011=2009>2008 | S  <0.024  2008<2009  2008=2011  2009=2011 |
| **Density of**  **adults**  **infected**  Density/100 m^2^ | 0.1 | 0.9 | 0.8 | S  <0.0001  α < β  α < γ  γ = β  β = γ > α | 0.3 | 1.5 | 0.7 | S  <0.002  α < β  α < µ  γ = β  β = γ > α | 0.1 | 0.4 | 1 | S  <0.0064  γ = β  β = α  γ > α | NS | NS | NS | NS |
