# Supplementary material for: Infection of Ixodes ricinus by Borrelia burgdorferi sensu lato in peri-urban forests of France
Source: PLoS One. 2017 Aug 28;12(8):e0183543. doi: 10.1371/journal.pone.0183543 (PMC5573218; doi:10.1371/journal.pone.0183543)
Supplement: S6 Table — (DOCX) [file pone.0183543.s006.docx]

Supplementary Table 6: Prevalence of *Borrelia* species identified in NC and C plots of Sénart forest

| **Nymphs** | **2008** | **2008** |  | **2009** | **2009** |  | **2011** | **2011** |  | **2008** | **2009** | **2011** |  |
| --- | --- | --- | --- | --- | --- | --- | --- | --- | --- | --- | --- | --- | --- |
|  | **C plots** | **NC plots** | **Statistics** | **C plots** | **NC plot** | **Statistics** | **C plots** | **NC plots** | **Statistics** | **total plots** | **total plots** | **total plots** |  |
|  | % | % | **2008** | % | % | **2009** | % | % | **2011** | % | % | % | **Statistics** |
|  |  |  | **C /NC** |  |  | **C /NC** |  |  | **C/NC** |  |  |  |  |
|  | 34.5 | 17.4 |  | 48.3 | 39 |  | 17 | 37 | S | 32.1 | 46.5 | 20.9 | S |
| ***B. afzelii*** |  |  | NS |  |  | NS |  |  | p<0.02 |  |  |  | <0.000003 |
|  |  |  |  |  |  |  |  |  | C < NC |  |  |  | 2009 > 2008 = 2011 |
|  | 22.5 | 52.2 | S | 28.7 | 34.1 |  | 30.4 | 29.6 |  | 26.7 | 29.8 | 30.2 |  |
| ***B. bss*** |  |  | <0.003 |  |  | NS |  |  | NS |  |  |  | NS |
|  |  |  | C < NC |  |  |  |  |  |  |  |  |  |  |
|  | 24.9 | 0 |  | 0.6 | 0 |  | 11.6 | 0 |  | 4.2 | 0.5 | 9.4 | S |
| ***B. garinii*** |  |  | NS |  |  | NS |  |  | NS |  |  |  | <0.005 |
|  |  |  |  |  |  |  |  |  |  |  |  |  | 2008 = 2011 > 2009 |
|  | 2.1 | 0 | NS | 1.1 | 2.4 |  | 0 | 11.1 |  | 1.8 | 1.4 | 2.2 | S |
| ***B. lusitaniae*** |  |  |  |  |  | NS |  |  | NS |  |  |  | <0.0002 |
|  |  |  |  |  |  |  |  |  |  |  |  |  | 2011 = 2008 > 2009 |
|  | 1.2 | 8.7 |  | 2 | 1 |  | 0 | 3 | S | 3 | 3 | 3 |  |
| ***B. spielmanii*** |  |  | NS |  |  | NS |  |  | <0.0004 |  |  |  | NS |
|  |  |  |  |  |  |  |  |  | C < NC |  |  |  |  |
|  | 12 | 8.7 |  | 6.3 | 2.4 |  | 12.3 | 11.1 |  | 11.5 | 5.6 | 12.2 |  |
| ***B. valaisiana*** |  |  | NS |  |  | NS |  |  | NS |  |  |  | NS |
|  |  |  |  |  |  |  |  |  |  |  |  |  |  |
|  | 2.8 | 0 |  | 4.6 | 12.2 |  | 5.4 | 0 |  | 2.4 | 6 | 4.3 |  |
| **Co-infection** |  |  | NS |  |  | NS |  |  | NS |  |  |  | NS |
|  |  |  |  |  |  |  |  |  |  |  |  |  |  |
|  | 12 | 4.8 | S | 14.5 | 8.5 | S | 9.7 | 5.6 | S | 10.0 | 12.8 | 8.5 | S |
| **Percentage of infected ticks** |  |  | <0.000006 |  |  | <0.001 |  |  | <0.007 |  |  |  | <0.0002 |
|  |  |  | C > NC |  |  | C > NC |  |  | C > NC |  |  |  | 2009 > 2008 = 2011 |
|  | B.a = B.bss = B.g > BV>=Bl=Bsp | B.bss = B.g = B.a >= B.v = B.sp = B.l |  | B.a > B.bss > Bg = Bv > Bsp = Bl | B.a = B.bss >= Bg = Bv = Bsp = Bl |  | Bbss = Bg = Ba = Bv >= Bl = Bsp | Ba = Bbss = Bg = Bv = Bsp >= Bl |  | B.a = B.bss = B.g > Bv = Bl >= Bsp | Ba >Bbss > Bg = Bv >= Bsp = Bl | Bbss = Ba = Bg >= Bv = Bl >= Bsp |  |

NS: non-significant; S: significant; C: plots with chipmunks, NC: plots without chipmunks
