# Supplementary material for: Infection of Ixodes ricinus by Borrelia burgdorferi sensu lato in peri-urban forests of France
Source: PLoS One. 2017 Aug 28;12(8):e0183543. doi: 10.1371/journal.pone.0183543 (PMC5573218; doi:10.1371/journal.pone.0183543)
Supplement: S7 Table — (DOC) [file pone.0183543.s007.doc]

Supplementary Table 7: Prevalence of *Borrelia* species in adult collected in 2008, 2009 and 2011 in α, β, γ plots of the Sénart forest

| **Adults** | **2008 2009 2011**  α **plots**  **%** | **2008 2009 2011**  β **plots**  **%** | **2008 2009 2011**  γ **plots**  **%** | **2008 2009 2011**  **Tot plots**  **%** | **Stat p**  α **/** β **/** γ | **2008**  **tot plots**  **%** | **2009**  **tot plot**  **%** | **2011**  **tot plots**  **%** | **Stat**  **years** |
| --- | --- | --- | --- | --- | --- | --- | --- | --- | --- |
| ***B.afzelii*** | **24** | 11.4 | 14 | **20** | NS | 5.4 | **31** | 21 | <0.0014  **2009>2008**  2009=2011  2008=2011 |
| ***B.bss*** | **28** | **31.4** | 15 | **28** | NS | **46** | **25** | 12 | <0.005  **2008>2011**  2008=2009  2009=2011 |
| ***B.garinii*** | 17 | **31.4** | 16 | **26** | NS | **24** | **29** | 24 | NS |
| ***B.lusitaniae*** | 0 | 0 | 0 | 0 | NS | 0 | 0 | 0 | NS |
| ***B.spielmanii*** | 3.4 | 0 | 8 | 7.3 | NS | 2.7 | 1.9 | 2.1 | <0.002  **2011>2009**  2011=2008  2008=2009 |
| ***B.valaisiana*** | **21** | **22.9** | 3 | 9.2 | NS | 16 | 9.6 | 18 | NS |
| **coinfection** | 6.9 | 2.9 | 3 | 4.9 | NS | 5.4 | 3.8 | 5.9 | NS |
| **Positif number** | 29 | 35 | 59 | 123 |  | 37 | 52 | 34 |  |
| **number analysed** | 287 | 221 | 718 | 1226 |  | 390 | 473 | 363 |  |
| **Percentage of infected ticks** | 10.1 | 16 | 8.2 | 10.03 | <0.004  β > γ  β = α  γ = α | 9.5 | 11 | 9.4 | NS |
| Statistics species | **Bbss=Ba=Bv=Bg** | **Bg=Bbss=Bv=Ba** | **Bg=B.bss=Ba** | **Bbss=Bg=Ba** |  | **Bbss=Bg=Bv** | **Ba=Bg=Bbss** | NS |  |
