# Supplementary figures and images for: Infection of Ixodes ricinus by Borrelia burgdorferi sensu lato in peri-urban forests of France
Source: PLoS One. 2017 Aug 28;12(8):e0183543. doi: 10.1371/journal.pone.0183543 (PMC5573218; doi:10.1371/journal.pone.0183543)

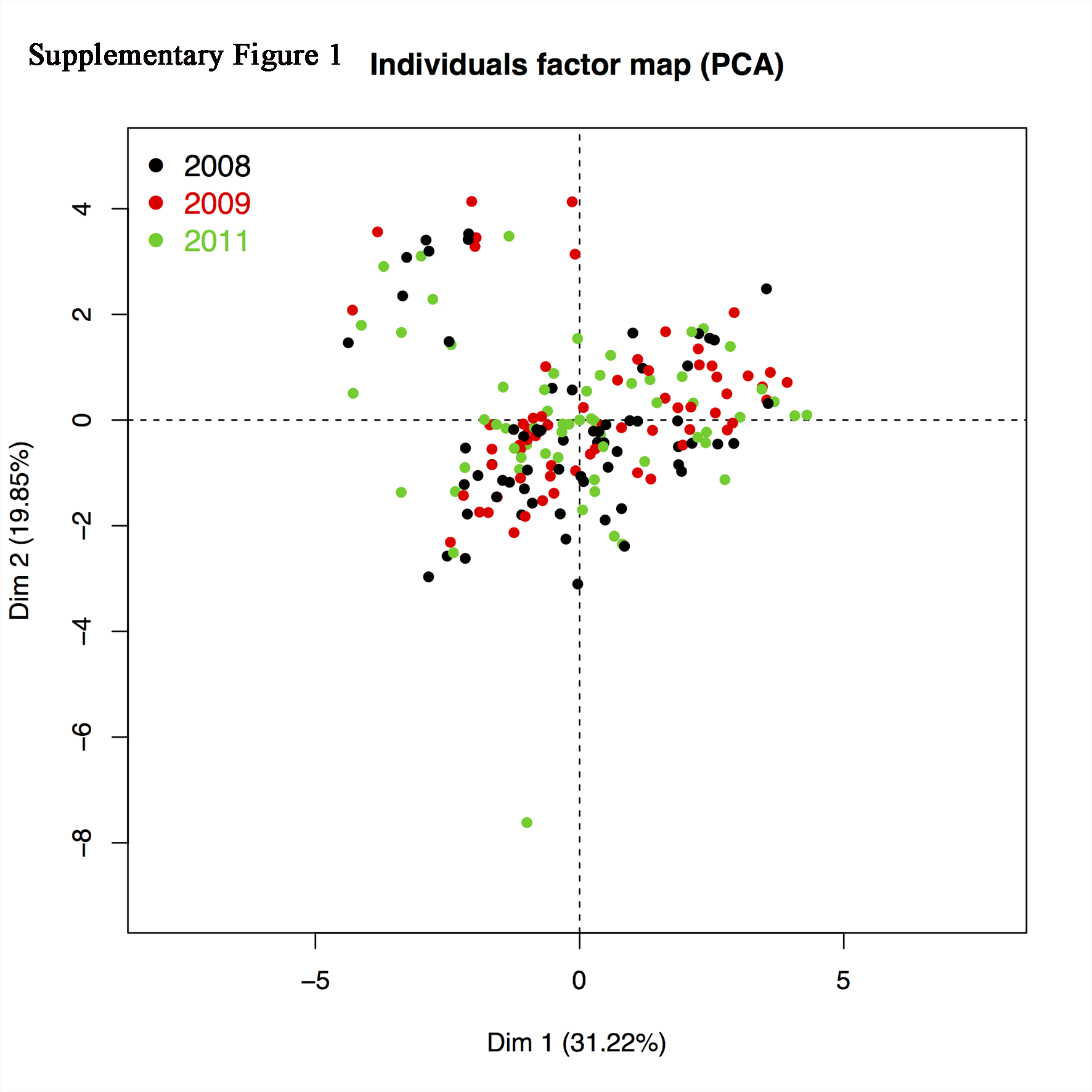

Supplement: S1 Fig — The dots are colored according year of the experiment. (TIF) [file pone.0183543.s008.tif]

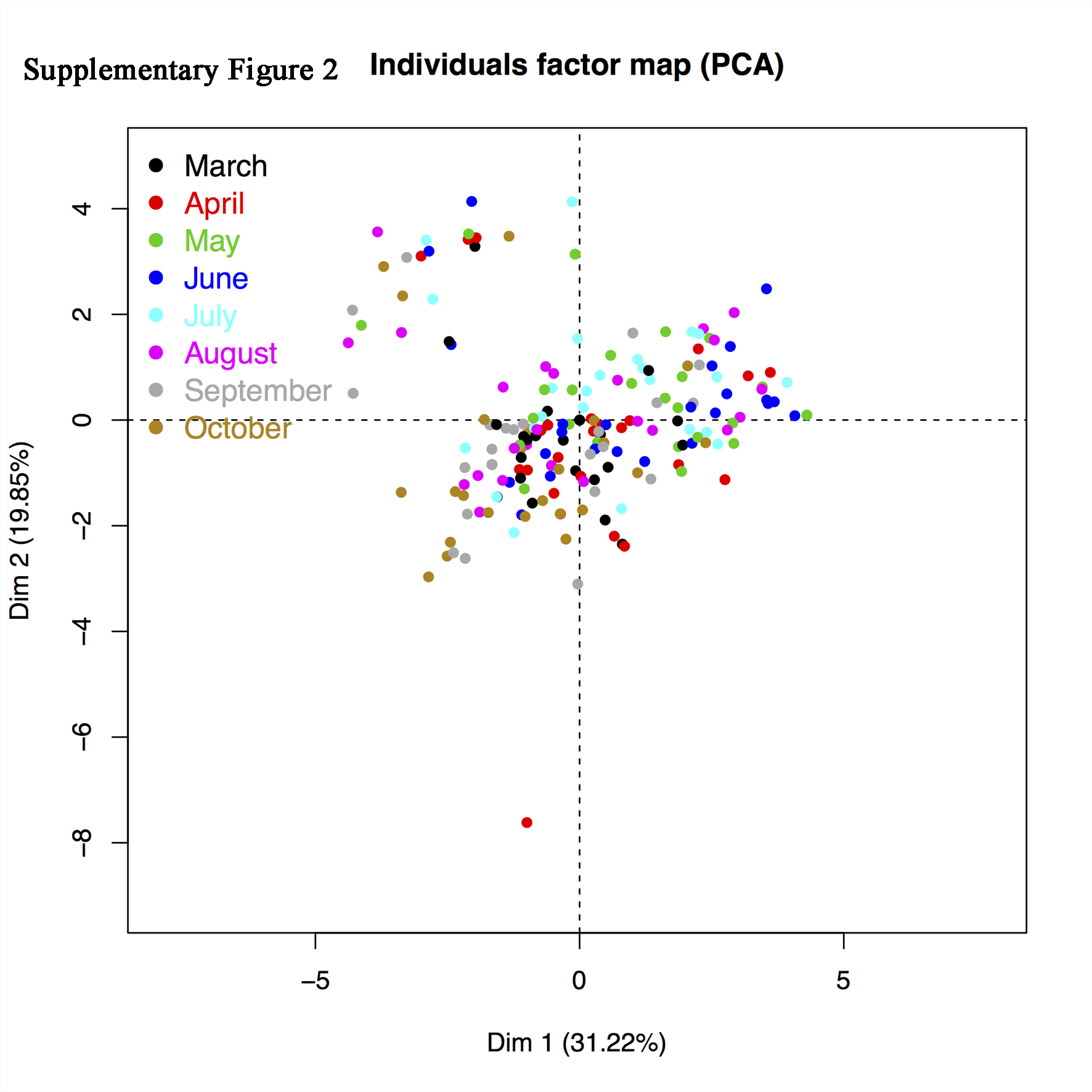

Supplement: S2 Fig — The dots are colored according to the month of the experiment. (TIF) [file pone.0183543.s009.tif]

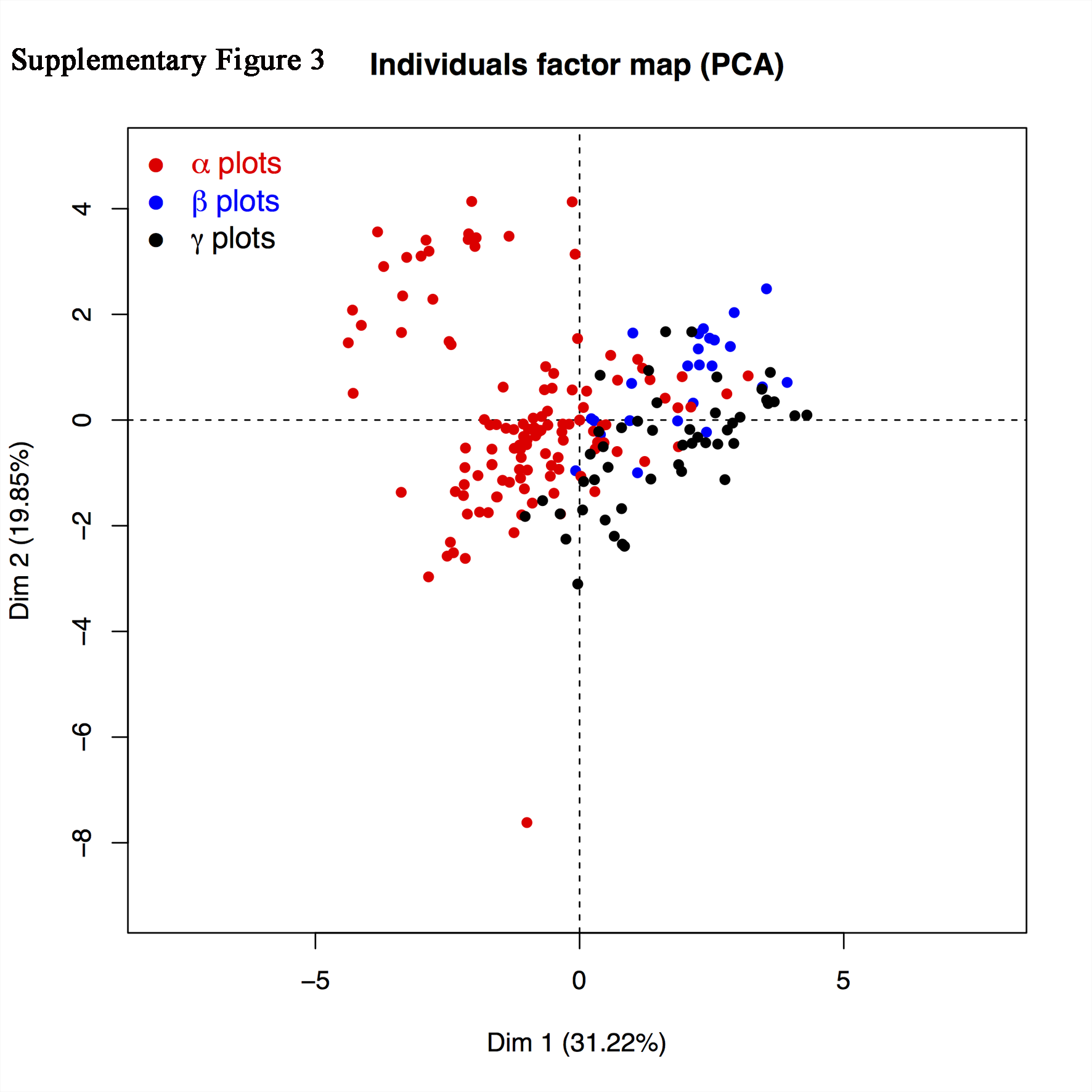

Supplement: S3 Fig — The dots are colored according to chipmunk abundance. (TIF) [file pone.0183543.s010.tif]
