# Supplementary material for: Infection of Ixodes ricinus by Borrelia burgdorferi sensu lato in peri-urban forests of France
Source: PLoS One. 2017 Aug 28;12(8):e0183543. doi: 10.1371/journal.pone.0183543 (PMC5573218; doi:10.1371/journal.pone.0183543)
Supplement: S4 Fig — The software used for drawing the tree was MEGA 5 (UPGMA method). (DOC) [file pone.0183543.s011.doc]

Supp Figure 4

**Group A**

*B.afzelii* one base difference to *B.afzelii* VS461

(T /C in position 56)

**Group B**

**Group D**

two bases differences with respect to B.afzelii VS461 (A / G in 60 et T / C in 161)

**Group E**

two bases differences to *B.afzelii* VS461

(T / C in 102 and A / T in 106)

**Group C**

one base difference to *B.afzelii* VS461

(T / C in 161)
