# Supplementary material for: Infection of Ixodes ricinus by Borrelia burgdorferi sensu lato in peri-urban forests of France
Source: PLoS One. 2017 Aug 28;12(8):e0183543. doi: 10.1371/journal.pone.0183543 (PMC5573218; doi:10.1371/journal.pone.0183543)
Supplement: S5 Fig — The software used for drawing the tree was MEGA 5 (UPGMA method). (DOC) [file pone.0183543.s012.doc]

***Group A***

three bases differences to B31 (T/– , A / –, C/T en 126)

***Group B***

***Group C***

three bases differences to B31

(G /in 22, in 30, T / A in 35, GC/AT in 54 55, - / T in 57, A / G in 70,A / G in 88 , G / A in 108, C/T in 148 158 and 164

Supplementary Figure 5
