# Supplementary material for: Infection of Ixodes ricinus by Borrelia burgdorferi sensu lato in peri-urban forests of France
Source: PLoS One. 2017 Aug 28;12(8):e0183543. doi: 10.1371/journal.pone.0183543 (PMC5573218; doi:10.1371/journal.pone.0183543)
Supplement: S6 Fig — The software used for drawing the tree was MEGA 5 (UPGMA method). (DOC) [file pone.0183543.s013.doc]

***Group A*** identical to PBi *B.bavariensis*

three bases differences to *B.garinii* 20047

(A / G in 26 A / G in 41 et G / A in 110)

***Group C***

***B.garinii***

three bases difference to *B. garinii* 20047

A / G in 26, A / G in 41 and T /C in 88

***Group D***

***B.garinii***

three bases difference to *B. garinii* 20047

A / G in 26, A / G in 41 and C/G in 90

***Group E***

***Group B***

four bases differences to *B.garinii* 20047

(A / G in 26, A / G in 41, G / A in 110 and A/T in 137)

Supplementary Figure 6
