# Supplementary material for: Infection of Ixodes ricinus by Borrelia burgdorferi sensu lato in peri-urban forests of France
Source: PLoS One. 2017 Aug 28;12(8):e0183543. doi: 10.1371/journal.pone.0183543 (PMC5573218; doi:10.1371/journal.pone.0183543)
Supplement: S7 Fig — The software used for drawing the tree was MEGA 5 (UPGMA method). (DOC) [file pone.0183543.s014.doc]

***Group A***

two bases differences with respect to *B.lusitaniae* Poti B2

G / T in 76 and C / T in 90

***Group B***

***B.lusitaniae***

four bases differences with respect to *B.lusitaniae* Poti B2

L30131.1 (C / T in 24, - /A, G /T and C / T in 80.

Supplementary Figure 7
