# Supplementary material for: Infection of Ixodes ricinus by Borrelia burgdorferi sensu lato in peri-urban forests of France
Source: PLoS One. 2017 Aug 28;12(8):e0183543. doi: 10.1371/journal.pone.0183543 (PMC5573218; doi:10.1371/journal.pone.0183543)
Supplement: S8 Fig — The software used for drawing the tree was MEGA 5 (UPGMA method). (DOC) [file pone.0183543.s015.doc]

***Group C***

four bases differences with respect to VS116 (C / A in 30, T / C in 37, C / T in 39, A / T in 62, C/T in 70 )

***Group B***

two bases differences with respect to VS116 (C / A in 30, C/T in70)

***Goup A***

Supplementary Figure 8
