# Supplementary material for: Infection of Ixodes ricinus by Borrelia burgdorferi sensu lato in peri-urban forests of France
Source: PLoS One. 2017 Aug 28;12(8):e0183543. doi: 10.1371/journal.pone.0183543 (PMC5573218; doi:10.1371/journal.pone.0183543)
Supplement: S9 Fig — The software used for drawing the tree was MEGA 5 (UPGMA method). (DOC) [file pone.0183543.s016.doc]

**Group A**

Eleven bases difference with respect to *B.spielmanii* PC-Eq17 N5

(T /- en, T / G in 17, T / G in 19, A / G in 32, Bloc GGCTT / AAACC 36 at 40, C / T in 48 et G / A in 110)

**Group B**

Two bases differences with respect to *B.spielmanii* PC-Eq17 N5 (G / T in 19 and G / A in 38)

Supplementary Figure 9
