# Supplementary material for: Infection of Ixodes ricinus by Borrelia burgdorferi sensu lato in peri-urban forests of France
Source: PLoS One. 2017 Aug 28;12(8):e0183543. doi: 10.1371/journal.pone.0183543 (PMC5573218; doi:10.1371/journal.pone.0183543)
Supplement: S10 Fig — The software used for drawing the tree was MEGA 5 (UPGMA method). (DOC) [file pone.0183543.s017.doc]

Supplementary Figure 10

**Group A**

***B.afzelii***

**Group B**

***B.afzelii*** identical to L30135.1 *B.afzelii* VS461

**Group D**

***B.afzelii***

**Group E**

***B.afzelii***

**Group B**

***B.garinii***

**Groupe A**

***B.burgdorferi*** sensu stricto

**Group A**

***B.garinii*** identical to PBi *B.bavariensis*
